# Chemical Profiling and In Vitro Cytotoxicity Assessment of Commercial Red Yeast Rice Ingredients

**DOI:** 10.3390/foods15162889

**Published:** 2026-08-18

**Authors:** Paola Nezi, Vittoria Cicaloni, Mattia Cicogni, Alessia Lucia Prete, Paolo Etiope, Barbara Barlozzini, Rita Pecorari, Laura Salvini, Laura Tinti

**Affiliations:** 1 Fondazione Toscana Life Sciences, Via Fiorentina 1, 53100 Siena, Italy; v.cicaloni@toscanalifesciences.org (V.C.); m.cicogni@toscanalifesciences.org (M.C.); alessialucia.prete@student.unisi.it (A.L.P.); paoloetiope@botanicalslab.it (P.E.); l.salvini@toscanalifesciences.org (L.S.); l.tinti@toscanalifesciences.org (L.T.); 2 Linneus Consulting, Via Giorgio Scalia 10 B, 00136 Roma, Italy; barbarabarlozzini@linneus.it (B.B.); ritapecorari@linneus.it (R.P.)

**Keywords:** untargeted metabolomics, red yeast rice, secondary metabolites, cytotoxicity, proteomics, bioinformatics, phytochemical characterization, food safety

## Abstract

Red yeast rice (RYR) food supplements are widely used for cholesterol management due to their monacolin content, particularly monacolin K (MK), which shares the same chemical structure as lovastatin, a compound associated with several side effects. The European Food Safety Authority (EFSA) highlighted uncertainties regarding the compositional variability, characterization of bioactive constituents, and safety of RYR products, concluding that a safe level of monacolin intake could not be established. In this study, twenty-seven commercial semi-processed RYR ingredients for food supplements manufacturing were investigated through a combined chemical and biological approach to characterize their composition and evaluate potential safety concerns. Targeted LC-MS/MS analyses revealed substantial variability in MK, monacolin hydroxy acid (MKA), and dehydromonacolin K (DMK) levels among products. Exploratory qualitative untargeted metabolomics enabled the putative annotation of multiple secondary metabolites, including azaphilones and polyketides, highlighting differences in chemical profiles. Correlation analyses indicated that DMK content was associated with pigment distribution and cytotoxic responses in human hepatocellular carcinoma cells and primary human skeletal muscle cells, whereas MK and MKA showed no significant association with toxicity under the tested conditions. Exploratory proteomic profiling suggested distinct protein-abundance patterns between RYR- and simvastatin-treated cells, providing hypothesis-generating information on functional annotations associated with these patterns. These findings emphasize the importance of comprehensive chemical characterization and quality control strategies considering both monacolins and secondary metabolites to improve the safety assessment of RYR supplements.

## 1. Introduction

Red yeast rice (RYR) is a traditional Chinese medicine and a widely used food supplement in East Asian countries [1,2]. It has been employed for centuries to treat digestive discomfort, circulatory disorders, and general weakness, and has more recently gained popularity in Western countries [3,4] as an alternative to standard statin therapy [5].

Its lipid-lowering properties are mainly attributed to monacolin K (MK), a structural analogue of lovastatin, which has been authorized in the EU and USA since 1987 for the treatment of hypercholesterolemia. MK and lovastatin interconvert between lactone and hydroxy acid forms, with the latter responsible for HMG-CoA reductase inhibition and downstream effects on cholesterol biosynthesis and LDL receptor regulation [6,7,8]. The MK/MKA ratio is pH-dependent, and whereas the hydroxy acid form naturally occurs in RYR, lovastatin requires in vivo hydrolysis as it acts as a prodrug [9]. Conversion may occur spontaneously or through CYP3A-mediated metabolism in the intestine and liver [9]. Despite their identical structure, MK and lovastatin exhibit distinct pharmacokinetic and bioavailability profiles.

Besides MK, RYR contains several other monacolins and a broad range of secondary metabolites including pigments, organic acids, sterols, decalin derivatives, flavonoids, lignans, coumarins, terpenoids and polysaccharides—most of which remain poorly characterized and only partially investigated for bioactivity [10].

In 2018, the European Food Safety Authority (EFSA) reassessed monacolin safety and reported that adverse events associated with RYR closely resemble those of lovastatin, based on data from WHO, ANSES, the Italian surveillance system and the FDA [6]. Between 2002 and 2018, 82 cases were reported to WHO Vigibase, mainly involving musculoskeletal disorders (including rhabdomyolysis), hepatic injury, and, to a lesser extent, neurological, gastrointestinal and dermatological effects [11]. Notably, adverse reactions were reported even at an intake of 3 mg/day, indicating the absence of a clear dose threshold below which effects do not occur. EFSA concluded that monacolin exposure in the range of 3–10 mg/day raises safety concerns and emphasized the substantial variability in monacolin content across RYR food supplements. This variability, together with the presence of other bioactive or potentially toxic secondary metabolites (e.g., citrinin) and the lack of full compositional characterization, contributes to substantial uncertainty in exposure assessment.

In its most recent opinion, EFSA stated that no sufficiently established safe threshold can be defined, even below 3 mg/day [12]. This conclusion is primarily driven by multiple, well-characterized sources of uncertainty identified by EFSA: (i) the substantial variability in the composition and relative abundance of monacolins in RYR-based food supplements, including the inconsistent ratio between monacolin K lactone and its hydroxy acid form; (ii) the widespread use of RYR in multi-ingredient botanical formulations, for which the safety of individual components and their combined effects has not been adequately evaluated; (iii) the lack of data on the bioactivity and toxicological relevance of RYR constituents other than monacolin K; (iv) uncertainties related to exposure in specific population groups, including pregnant and lactating women and breast-fed infants, for whom safety cannot be assessed; and (v) the potential for clinically relevant interactions, particularly with substances inhibiting CYP3A4, as well as unknown interactions with other ingredients present in complex formulations. Collectively, these uncertainties prevent a reliable characterization of exposure and hazard, thereby precluding the establishment of a health-based guidance value according to EFSA risk assessment principles.

In this context, the present study was designed to address the major knowledge gaps highlighted by EFSA. Firstly, we conducted a detailed chemical characterization of commercial RYR supplements, quantifying monacolins and defining MK/MKA ratios using LC-UV and high-resolution MS. We then applied an untargeted metabolomics approach to profile the broader landscape of secondary metabolites contributing to RYR’s chemical complexity.

Finally, cytotoxicity assays were complemented by an exploratory proteomic comparison of RYR- and simvastatin-treated cells to generate hypotheses regarding potentially different cellular response patterns. This integrated analytical and in vitro toxicological approach provides new insights into compositional features potentially relevant to the safety of RYR ingredients and contributes to generating experimental evidence supporting their more comprehensive compositional and in vitro safety characterization.

## 2. Materials and Methods

### 2.1. General Materials

Monacolin K (MK) reference standard was purchased from PhytoLab GmbH & Co. KG (Vestenbergsgreuth, Germany). LC-MS grade acetonitrile, formic acid, methanol, ethanol, and ultrapure water were purchased from Merck Group.

Twenty-seven commercial RYR ingredients with different declared monacolin contents were kindly provided by different companies as detailed in Table 1. Each material was assigned an anonymized sample identifier. All samples were sourced from operators based in Italy; however, these operators did not necessarily correspond to the original manufacturers of the RYR ingredients. The raw materials were reported by the suppliers to originate from different geographical areas, reflecting the global supply chain of RYR-based ingredients. However, precise information on the country of origin was not consistently available and could not be independently verified.

All samples were supplied as semi-processed milled RYR materials intended for use as ingredients in food supplement manufacturing and were not finished consumer products. Batch information was available for several samples but is not disclosed due to confidentiality agreements with the suppliers. Moreover, multiple batches of the same commercial material were not systematically included. Consequently, the present sampling design did not allow an assessment of batch-to-batch variability. Samples were stored at 25 °C under controlled humidity in an ISO 9001:2015-certified facility [13] (certificate Q/1765/24), ensuring standardized workflows, traceability, and quality control in line with the storage requirements for semi-processed herbal materials used in food supplement manufacturing. According to the manufacturers’ quality certificates, citrinin levels in all samples were below the EU limit of 100 μg/kg.

Overall, the sampling strategy reflects the variability among commercial RYR ingredients supplied to the Italian market; however, the limited sample size and the lack of systematic geographical and batch stratification should be considered when interpreting the generalizability of the results.

### 2.2. Targeted Quantification of Monacolins

A standard stock solution was prepared by dissolving 10 mg of MK reference standard in 1 mL of ethanol. Working solutions were obtained by diluting aliquots in 75% ethanol to yield five calibration levels (0.5 to 10 ppm). The calibration curve was constructed by plotting peak area versus concentration, showing good linearity over the tested range (R^2^ = 0.9978). The limit of detection (LOD) and limit of quantification (LOQ), calculated from the standard deviation of the response and the slope of the calibration curve, were 0.11 ppm and 0.33 ppm, respectively. Precision was evaluated in terms of repeatability (intra-day) and intermediate precision (inter-day) by analyzing calibration standards in triplicate over three consecutive days (*n* = 9). Relative standard deviations (% RSD) were below 10% for all calibration levels, indicating acceptable method precision. Accuracy and precision were further assessed using independently prepared quality-control solutions at 0.75, 3, and 7 ppm. The mean measured concentrations correspond to accuracy from 98 to 100%, with coefficients of variation below 10%.

For sample preparation, 200 mg of powder was extracted in 15 mL of 75% ethanol, sonicated for 45 min, and centrifuged at 12,000 rpm for 10 min at 22 °C. The supernatant was transferred into clean vials. Due to the wide concentration range of analytes, extracts were analyzed undiluted and after 1:25 and 1:50 dilutions. Dilution integrity was verified for all samples by comparing the back-calculated concentrations obtained at the different dilution levels after correction for the corresponding dilution factors. Each sample was analyzed in triplicate, with duplicate injections.

Samples were freshly prepared before analysis. Quality-control solutions were injected after every nine sample analyses to monitor analytical performance and short-term MK stability throughout the sequence. No relevant variations in the measured QC concentrations were observed during the analytical runs. Quantification of monacolins was performed using an Ultimate 3000 UPLC system (Thermo Fisher Scientific Inc., Waltham, MA, USA) coupled with a diode array detector (237 nm) and a high-resolution Q-Exactive Plus Hybrid Quadrupole–Orbitrap™ mass spectrometer (Thermo Fisher Scientific Inc., Waltham, MA, USA) operating in positive electrospray ionization mode. Targeted analysis was performed by Parallel Reaction Monitoring (PRM) scan. Analyte-specific precursor ions were isolated and fragmented using optimized collision energies to generate characteristic product ions for unequivocal identification.

Chromatographic separation was achieved on an Acquity UPLC BEH C18 (Waters Corporation, Milford, MA, USA) column (2.1 mm × 15 cm, 1.7 µm, Waters). The mobile phase consisted of water with 0.1% formic acid (A) and acetonitrile with 0.1% formic acid (B). The gradient started at 35% B for 1 min, increased to 75% B over 19 min, held for 5 min, and then re-equilibrated to initial conditions. Flow rate was maintained at 0.220 mL/min, injected volume was 10 µL, and column temperature was kept constant at 40 °C.

Potential chromatographic interference was assessed by monitoring the chromatograms at 237 nm and at additional wavelengths characteristic of RYR pigments. No pigment-related co-eluting peaks were observed at the retention time of MK. Retention time, accurate mass, and high-resolution MS/MS spectra were used for analyte identification. Quantification was performed using the MK calibration curve recorded prior to sample analysis. Concentrations obtained from the calibration curve were corrected for the extraction volume and dilution factor and expressed as percentage by weight relative to the initial sample mass. Due to the lack of certified reference standards for other monacolins, their concentrations were estimated using the MK calibration curve and expressed as MK equivalents. Therefore, these values should be considered semi-quantitative and interpreted comparatively rather than as absolute concentrations.

### 2.3. Statistical Analysis

The targeted monacolin dataset was imported into the MetaboAnalyst 6.0 online platform [14]. Hierarchical clustering analysis (HCA) was performed using Euclidean distance and complete linkage to visualize compositional similarities among RYR ingredients. To avoid redundant weighting of mathematically complementary variables, MKA/(MK + MKA) was excluded from the HCA, which was therefore based on MK/(MK + MKA), DMK/(MK + MKA), and MSEC/(MK + MKA). The HCA was considered exploratory and was not intended as a formal compositional data analysis.

Pearson correlation analysis was performed separately using the original semi-quantitative metabolite levels rather than the shared-denominator ratios. Pairwise correlations among monacolins and pigment measurements were evaluated across the samples (N = 27). To account for multiple hypothesis testing, *p*-values were adjusted using the Benjamini–Hochberg false discovery rate (FDR) procedure, considering significance at an adjusted q-value < 0.05.

### 2.4. Metabolomics Untargeted Analysis

Exploratory qualitative metabolic profiling of RYR samples was performed using an Ultimate 3000 UPLC system coupled to a Q-Exactive Plus Hybrid Quadrupole–Orbitrap™ mass spectrometer (Thermo Fisher Scientific). The analysis was intended to provide broad qualitative chemical coverage and was not designed for absolute or relative metabolite quantification.

Three 100 mg aliquots of each powdered sample were extracted with 10 mL of 75%, 50%, and 30% ethanol by ultrasonication for 45 min. Extracts were centrifuged at 13,000 rpm for 10 min, and the supernatants were collected, filtered (0.22 µm), and injected into the LC–HRMS system without further dilution. All analyses were performed in technical triplicate.

Data were acquired in both positive and negative electrospray ionization (ESI) modes over a mass range of *m*/*z* 200–2000. HR-MS parameters were: spray voltage 3.5 kV (positive) and 3.0 kV (negative), sheath gas 20 arbitrary units, auxiliary gas 5 arbitrary units, capillary temperature 320 °C, and resolution 35,000. Full scan MS/dd-MS2-Top N acquisition was used, with ion selection for fragmentation based on abundance. MS/MS spectra were generated by Higher Energy Collision Dissociation (HCD) at 30 eV, with a mass accuracy threshold of 5 ppm.

Chromatographic separation was performed on an ACQUITY UPLC BEH C18 column (2.1 × 150 mm, 1.7 µm; Waters Corporation, Milford, MA, USA) column (2.1 × 150 mm, 1.7 µm; Waters) at 35 °C. Mobile phases were 0.1% formic acid in water (A) and 0.1% formic acid in acetonitrile (B). The gradient started at 2% B for 1 min, increased to 100% B over 50 min, held for 2 min, and returned to initial conditions. The flow rate was 0.2 mL/min, and the injection volume was 10 µL.

Raw LC–MS/MS data were processed using Compound Discoverer 3.3 (Thermo Fisher Scientific) [15]. Feature detection and alignment were performed with default settings, except for a retention-time tolerance of 0.2 min and a mass tolerance of 10 ppm. Solvent blanks were analyzed under identical conditions to exclude background peaks. The data processing workflow included automated feature detection, chromatographic alignment, background subtraction, isotope/adduct grouping, and database-assisted annotation. For each detected feature, CD returned the compound name, molecular formula, precursor *m*/*z*, calculated molecular weight, retention time (RT), maximum peak area, ionization mode (ESI positive or negative), and MS/MS-based annotation obtained through matching against the spectral and structural databases integrated into the platform (mzCloud, mzVault, ChemSpider, Mass List, Metabolika). The resulting filtered feature lists are provided in Supplementary Materials. An in-house screening tool was applied to filter detected features according to the following criteria:

- DATABASE (*m*/*z* Cloud, *m*/*z* Vault, Metabolika, ChemSpider, Mass List): compounds with full or partial matches in at least one of the five specified databases were retained. For instance, even a single partial match in ChemSpider dictated the inclusion of a compound, ensuring comprehensive informative coverage.
- DELTA ppm (peak recognition error): A range of −3/+3 ppm was adopted to preserve compounds with peak recognition errors within acceptable limits.
- RT (retention time): The retention time selection was customized based on the specific requirements of the method, permitting the retention of compounds within a timeframe of 5 min to 50 min. However, flexibility was provided to select a range from 0 min to 2 h, ensuring greater adaptability to the experimental context.
- AREA (Max): Areas were sorted in descending order, and a modifiable threshold was introduced to select the range of areas for exclusion. An option to exclude compounds with areas below 10^−5^ was provided, optimizing the management of low-relevance information.
- MS2 (tandem mass spectrometry): only features for which MS/MS fragmentation data were available were retained in the final annotated list.

The resulting dataset was cross-checked against the published literature to support and contextualize the putative annotations within known phytochemical RYR profiles. For the structural classification of metabolites, we employed Graph Isomorphism Networks (GINs), following the framework described in [16,17] to assign each putatively annotated metabolite to its respective structural class. This tool enhances the precision and accuracy of metabolite classification, providing valuable insights into the structural diversity of the putatively annotated compounds. In accordance with the Metabolomics Standards Initiative (MSI), metabolites confirmed with authentic reference standards and MS/MS fragmentation matching were classified as MSI Level 1; features annotated on the basis of accurate mass and MS/MS spectral similarity to database entries are classified as MSI Level 2, and unannotated or partially characterized features are assigned to MSI Levels 3/4. The MSI confidence level for each feature is explicitly indicated in Supplementary Materials. Non–standard-confirmed metabolites discussed in the main text should be regarded as putative annotations (MSI Level 2) pending further validation with authentic standards.

### 2.5. Preparation and Characterization of MKA, DMK and RYRlowDMK Fractions

A separate standardized commercial red yeast rice ingredient, RICE-KOLIN™ containing 5% monacolin K, batch E2024112802/R1, was supplied by Nutraceutica S.r.l. (Monterenzio, Bologna, Italy) and was used exclusively for the preparative isolation of MKA, DMK and RYRlowDMK fractions. According to the supplier’s certificate of analysis, the ingredient was manufactured on 28 November 2024 and consisted of a concentrated ethanolic extract obtained from *Oryza sativa* L. caryopses fermented with *Monascus purpureus* Went., subsequently spray-dried and re-adsorbed onto red yeast rice. The declared MK content was 50.14 mg/g and the residual moisture content was 5.68%. The certificate of analysis also reported compliance with the applicable specifications for heavy metals, pesticides, polycyclic aromatic hydrocarbons, citrinin, total microbial count, and pathogens, with aflatoxin B1 below 5 μg/kg and total aflatoxins below 10 μg/kg. The material was stored in its original container in a cool, dry place protected from light. All subsequent operations were performed under light-protected conditions because of the photolability of the conjugated diene system of the monacolin hexahydronaphthalene core. A 5.00 g aliquot of RICE-KOLIN™ was suspended in 50 mL of 75% ethanol in water (*v*/*v*), corresponding to a solid-to-liquid ratio of 1:10 (*w*/*v*), and extracted in an ultrasonic bath at 40 °C for 45 min. The suspension was vacuum-filtered through 10 μm filter paper using a Büchner funnel. The filtrate was evaporated to dryness under reduced pressure using a rotary evaporator with the water bath maintained at 50 °C and a pressure of 100 mbar. The resulting dry residue, weighing 1.09 g, was redissolved in 10 mL of 75% ethanol in water (*v*/*v*) and filtered through a 0.45 μm membrane before preparative HPLC fractionation.

Preparative fractionation was performed using a Nexera Prep HPLC (Shimadzu Corporation, Kyoto, Japan) system equipped with a Sepachrom Adamas C18-XBond column (5 μm, 250 × 10 mm), maintained at 35 °C. The mobile phases were ultrapure water (A) and acetonitrile (B). The following gradient was applied: 35% B from 0 to 1.5 min; 35–75% B from 1.5 to 19 min; 75% B from 19 to 21 min; 75–35% B from 21 to 22 min; and 35% B from 22 to 25 min. The flow rate was 4.0 mL/min, the injection volume was 20 μL, and UV detection was performed at 237 nm with a bandwidth of 4 nm. Fractions were collected on a time basis using the collection windows reported: MKA—14.50, 15.00 min; DMK—23.00, 23.75 min.

In parallel with the collection of the individual MKA and DMK fractions, a composite RYR fraction with reduced DMK content (RYRlowDMK) was prepared by collecting the chromatographic eluate from 2.0 min to the end of monacolin K elution at 18.50 min. The subsequent chromatographic portion was discarded. This late-eluting portion contained DMK together with other more strongly retained constituents. Therefore, RYRlowDMK should not be interpreted as a selectively DMK-depleted preparation, but rather as a chromatographically fractionated RYR preparation characterized by reduced DMK content together with depletion of other late-eluting constituents. The procedure was repeated over 50 consecutive injections and the corresponding fractions were pooled. The pooled MKA, DMK, and RYRlowDMK fractions were taken to dryness under a stream of nitrogen, avoiding heating, and stored at −20 °C protected from light until use. Following preparative fractionation, the fractions were characterized by LC-HRMS using the targeted analytical method described in Section 2.2. Chromatographic assignments were evaluated on the basis of accurate mass, retention time, and MS/MS evidence, where available. The monacolin profile of RYRlowDMK was specifically assessed to determine the relative abundance of MK, MKA, DMK, and the other monitored monacolins and to characterize the compositional changes introduced by the fractionation procedure.

Chromatographic purity of the preparatively obtained fractions was assessed by UV peak-area normalization at 237 nm. The MKA fraction accounted for approximately 97% of the total integrated UV signal, whereas DMK accounted for >99%, with no additional UV-detectable peaks observed under the applied analytical conditions.

### 2.6. Cytotoxicity Assay

Viability assays were performed in human hepatocellular carcinoma cells (HepG2) and primary human skeletal muscle cells (HSkMC). HepG2 cells were seeded in 48-well plates (1 × 105 cells/well) and cultured in Dulbecco’s Modified Eagle Medium (DMEM; Life Technologies Corporation, Carlsbad, CA, USA) supplemented with 10% fetal bovine serum (FBS) and antibiotics at 37 °C and 5% CO_2_. After 48 h, cells underwent a starvation step by replacing the medium with DMEM containing 2% FBS for 3 h. Cells were then exposed for 24 h to different concentrations (0.1–50 μM) of simvastatin, atorvastatin, monacolin K standard, or six selected RYR extracts. Extracts were prepared in ethanol, sonicated, and centrifuged to remove insoluble material. The resulting supernatants were directly diluted in cell-culture medium. Treatment concentrations were expressed in µM based on the quantified monacolin K content in each extract. Because RYR extracts were normalized according to their monacolin K content, the corresponding total extract concentrations varied among samples depending on their individual monacolin K titres (see Supplementary Table Amount Extract). The extract concentrations corresponding to each monacolin K treatment concentration were calculated from the measured monacolin K content of each extract.

This normalization allowed treatments to be administered at equivalent MK concentrations, enabling assessment of the contribution of additional metabolites (including DMK and secondary monacolins) to cytotoxic variability across samples.

Alamar Blue reagent (final concentration 0.15 mg/mL; Merck KGaA, Darmstadt, Germany) was added to each well, and cells were incubated for 3 h under standard culture conditions. Fluorescence of reduced Alamar Blue was measured at 560/590 nm (excitation/emission) using a microplate reader. Cell viability was expressed as a percentage relative to the corresponding vehicle control. The final ethanol concentration in the culture medium was approximately 0.5% (*v*/*v*), and the corresponding vehicle-control wells contained the same ethanol concentration. To assess potential direct interference of the RYR extracts with the Alamar Blue assay, cell-free controls were performed for each extract under the same experimental conditions used for the cell-based assay. Briefly, each extract was incubated with culture medium and Alamar Blue reagent in the absence of cells. A cell-free background control containing culture medium and Alamar Blue reagent, without RYR extract or cells, was included in parallel. Signals obtained from the extract-containing cell-free controls were compared with the corresponding background control to assess potential extract-dependent interference with the Alamar Blue readout.

Statistical analysis was performed using one-way analysis of variance (ANOVA), followed by Dunnett’s post hoc test for multiple comparisons between each treatment condition and the corresponding control. A *p*-value < 0.05 was considered statistically significant. Three independent biological experiments were performed using separately prepared cell cultures, with each condition assessed in technical triplicate. For each biological experiment, the technical triplicates were averaged to obtain a single value per condition. Statistical analyses were therefore performed using the three independent biological experiments as the experimental units (n = 3).

Primary human skeletal muscle cells (HSkMC; ATCC PCS-950-010, batch 81202212) were obtained from the thigh tissue of a Caucasian female neonate. The cells were supplied at passage 3 and were used between passages 3 and 7. HSkMC were cultured at 37 °C and 5% CO_2_ in mesenchymal stem cell basal media (ATCC PCS-500-030) supplemented with primary skeletal muscle growth kit (ATCC PCS-950-040) containing L-glutamine (10 mM), rhEGF (5 ng/mL), dexamethasone (10 μM), rhFGF-b (5 ng/mL), rh insulin (25 μg/mL), 4% FBS, and antibiotics. Once the HSkMC reached 80–90% confluence, they were cultured in 48-well plates (4 × 10^4^ cells/well). Treatments with simvastatin, atorvastatin, monacolin K, and six RYR extracts (0.1–50 μM) were carried out for 24 h under the same conditions used for HepG2 cells. Extract concentrations were normalized to monacolin K content as described above, allowing evaluation of additional metabolites’ contribution to cytotoxicity. Viability was assessed using Alamar Blue as described above, with fluorescence measured at 560/590 nm. The results were expressed as a percentage of viable cells in relation to the corresponding vehicle control. Data are expressed as mean ± SD, and a *p*-value < 0.05 was considered statistically significant. The corresponding vehicle control was included in all experiments. Statistical analysis was performed using one-way analysis of variance (ANOVA), followed by Dunnett’s post hoc test for multiple comparisons to evaluate differences between experimental groups and the control group. Three independent biological experiments were performed on different experimental days using separately prepared cell cultures, with each condition assessed in technical triplicate. In the Supplementary Materials, cytotoxicity raw files were reported.

### 2.7. Proteomic Analysis

For proteomic analysis, HepG2 cells (passages 12–16) and HSkMC cells (passages 3–7) were seeded in 6-well plates at densities of 6 × 10^5^ and 1 × 10^5^ cells/well, respectively. Before treatment, HepG2 cells underwent a 3 h starvation step in DMEM supplemented with 2% FBS, whereas HSkMC cells were differentiated for 12 days using skeletal muscle differentiation medium (ATCC PCS-950-050). Cells were subsequently exposed for 48 h to the selected low-DMK RYR ingredient MON-MK-63-34, normalized to an MK-equivalent concentration of 25 µM, 25 µM simvastatin, or the corresponding vehicle control. MON-MK-63-34 was selected because it was naturally characterized by a very low DMK content and did not significantly affect cell viability under the tested conditions. The experimental groups were defined as follows:

- Control (Ctrl), cells maintained under the corresponding vehicle-control conditions;
- Simvastatin (SIM), cells treated with 25 µM simvastatin to characterize the proteomic response to simvastatin exposure;
- Red Yeast Rice (RYR), cells treated with MON-MK-63-34 at an MK-equivalent concentration of 25 µM to characterize the proteomic response to RYR exposure.

For each cell type and experimental condition, three independent biological cell cultures were prepared and treated separately. Following treatment, the three biological samples corresponding to each condition were pooled to generate one composite biological sample per condition. Each pooled sample was subsequently divided into three aliquots, which were independently processed using the SP3 workflow, including protein digestion, and analyzed by LC–MS/MS. These three preparations were considered technical replicates because they originated from the same pooled biological sample. Thus, although each pooled sample incorporated material from three independently treated biological cultures, pooling prevented estimation of inter-sample biological variability. The technical replicates therefore provided information on the reproducibility of sample preparation and LC–MS/MS analysis but were not considered independent biological observations.

Proteomic profiling of HepG2 and HSkMC was performed using a shotgun LC–MS/MS workflow followed by bioinformatic analysis. Protein extraction was carried out using single-pot, solid-phase enhanced sample preparation (SP3) [18]. Cells were lysed in cold radioimmunoprecipitation buffer (Thermo Fisher Scientific Inc., Waltham, MA, USA), and protein concentration was determined by BCA assay (Thermo Fisher Scientific Inc., Waltham, MA, USA).

For each sample, 25 µg of protein was adjusted to 50 µL with Milli-Q water and reduced with 5 µL of 10 mM dithiothreitol for 1 h at 60 °C. Alkylation was performed in the dark with 1.25 µL of 25 mM iodoacetamide for 45 min. SP3 SpeedBead magnetic particles (Cytiva, Marlborough, MA, USA) were washed in absolute ethanol, resuspended, and added to samples together with an equal volume of ethanol to promote protein binding. Samples were incubated for 15 min at 1000 rpm, and the supernatant was removed using a magnetic rack. Beads were washed three times with 80% ethanol to eliminate detergents and contaminants. Proteins bound to the beads were digested overnight at 37 °C with Trypsin Gold (Promega Corporation, Madison, WI, USA) at a 1:50 enzyme-to-protein ratio in 50 mM ammonium bicarbonate. Peptides were recovered by centrifugation (13,800× *g*, 10 min), desalted using OASIS cartridges (Waters Corporation, Milford, MA, USA), dried, and reconstituted in 0.1% formic acid to a final concentration of 1 mg/mL.

Peptide mixtures were analyzed by LC–MS/MS using a Q Exactive™ HF-X hybrid quadrupole–Orbitrap™ mass spectrometer (Thermo Fisher Scientific Inc., Waltham, MA, USA). Chromatographic separation was achieved on a PepMap™ RSLC C18 column (75 µm × 500 mm, 2 µm, 100 Å) at 35 °C and 300 nL/min. Mobile phases were 0.1% formic acid in water (A) and 0.1% formic acid in 80% acetonitrile (B). The gradient started at 5% B for 5 min, increased to 90% B over 97 min, held for 9 min, and then returned to initial conditions. Data-dependent acquisition was used, selecting the 12 most intense ions from each full MS scan (200–2000 *m*/*z*) for higher-energy collisional dissociation (HCD) fragmentation.

Protein identification was performed with Proteome Discoverer 2.5 (Thermo Fisher Scientific Inc., Waltham, MA, USA) using the Sequest search engine against the Homo sapiens UniProtKB database (taxonomy ID 9606; 83,427 entries, January 2024). Search parameters specified: trypsin digestion with up to 2 missed cleavages; dynamic modifications of methionine oxidation (+15.995 Da) and N-terminal acetylation (+42.011 Da); static modification of carbamidomethylation on cysteine (+57.021 Da). Target-decoy search validation applied a strict false discovery rate (FDR) threshold of <0.01 (1%) at both the peptide and protein levels. Protein identification required a minimum criterion of ≥2 unique peptides per protein. Bioinformatic and statistical processing were performed using Perseus (v1.6.15.0). Quantified proteins were filtered to retain features present in at least 70% of samples within at least one treatment group. Missing values were imputed from a normal distribution (width = 0.3, downshift = 1.8).

Protein-abundance profiles were compared across experimental conditions on an exploratory basis. Complete processed protein-level results for both HepG2 and HSkMC cells, including individual protein-abundance values, pairwise log2 fold changes, ANOVA *p*-values, FDR-adjusted ANOVA q-values, and Tukey HSD significant-pair assignments, are provided in the Supplementary Material. Statistical outputs derived from the technical replicates were retained only as descriptive measures of technical reproducibility, encompassing both sample preparation and LC–MS/MS analysis, and were not interpreted as estimates of biological variability or as confirmatory evidence of differential protein expression. Proteins showing an absolute |log_2_ fold-change| ≥ 0.58 were selected for exploratory protein–protein interaction and functional-enrichment analyses.

Protein–protein interaction networks and functional enrichment analyses were conducted using STRING v12.0 [19], with a confidence threshold of 0.7 to retain high-confidence associations. STRING integrates experimental evidence and computational predictions and includes both direct physical interactions and indirect functional associations. STRING-reported FDR-adjusted *p*-values were used to characterize and rank over-represented functional terms within the selected protein sets. These enrichment statistics refer to over-representation within the exploratory protein sets and were not interpreted as evidence of biologically replicated pathway activation.

The mass spectrometry proteomics data have been deposited with the ProteomeXchange Consortium via the PRIDE partner repository [20] under dataset identifier PXD082271.

## 3. Results

### 3.1. Quantification of Monacolins

The first analytical step consisted of quantifying the total monacolin content of commercial RYR ingredients. Under optimized conditions, MK eluted at approximately 20.3 min. High resolution mass spectrometry (HRMS) and MS/MS were used to confirm monacolins’ identities based on accurate mass and spectral matching. A method including Parallel Reaction Monitoring (PRM) was implemented to enhance selectivity.

Table 2 summarizes the monitored monacolins, including chemical formula, precursor ions [M+H]+, and retention time observed for each monacolin. Acidic monacolins M (MMA), J (MJA), and X (MXA) were included in the PRM mass list, but none of these species were detected in any analyzed samples.

**Figure 1:**
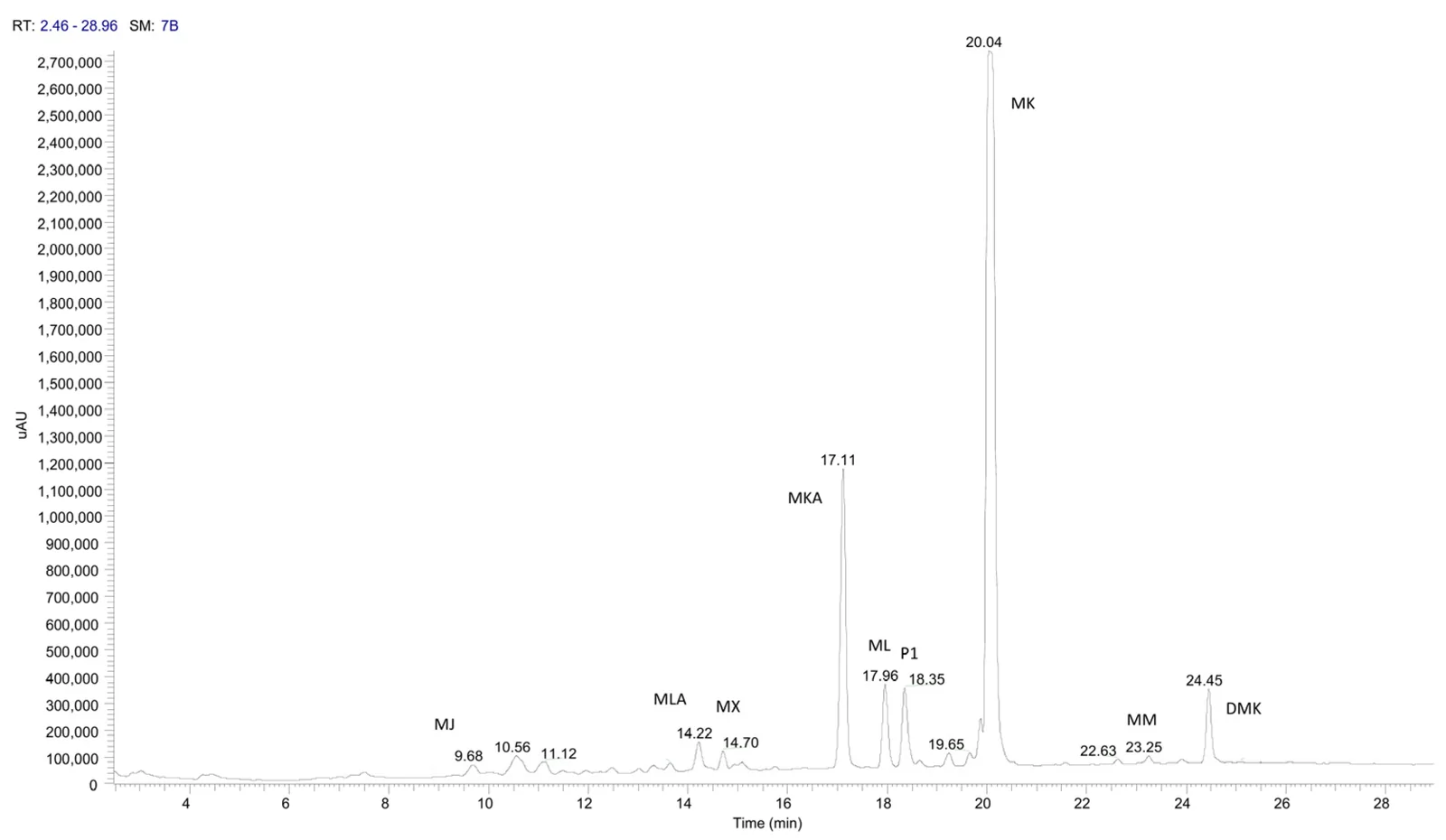
LC-UV chromatogram acquired at a wavelength of 237 nm.

A representative LC-UV chromatogram acquired at 237 nm was shown in Figure 1.

Once the retention time of each monacolin had been conclusively assigned, twenty-seven RYR ingredients were analyzed. According to product documentation, the declared monacolin contents ranged from 0.4 to 5%, when available (NA).

Quantification was performed by monitoring absorbance at 237 nm and corroborated using mass spectrometric data. The amounts of MK, acidic monacolin K (MKA), dehydromonacolin K (DMK), and the sum of secondary monacolins (MSEC) were calculated using the external calibration curve of MK and expressed as MK equivalents. Therefore, these values are semi-quantitative and intended for comparative purposes rather than absolute determination. All measurements were performed in triplicate, and results are reported as mean ± SD in Table 3 as % *w*/*w*, together with declared values.

Measured total monacolins (TOT) ranged from 0.45 to 7.84% (*w*/*w*). In most ingredients, experimental values were consistent with declared contents. However, several samples (see “*” Table 3) showed discrepancies from label claims, which may reflect variability in raw material composition or formulation differences. The declared titre was used as the nominal reference for this comparison. The analysis was intended to characterize the individual commercial ingredients included in the study and was not designed to assess batch-to-batch variability.

### 3.2. Multivariate Statistical Analysis

To investigate compositional variability among products, semi-quantitative monacolin data were expressed as relative ratios to MK + MKA (Table 4). Across samples, MK/(MK + MKA) ranged from 0.72 to 0.99, MKA/(MK + MKA) from 0.01 to 0.28, DMK/(MK + MKA) from 0.00 to 0.21, and MSEC/(MK + MKA) from 0.00 to 0.23. Because MK/(MK + MKA) and MKA/(MK + MKA) are mathematically complementary variables, MKA/(MK + MKA) was excluded from the Hierarchical Cluster Analysis (HCA) to avoid redundant weighting of the same compositional relationship. HCA was therefore performed using MK/(MK + MKA), DMK/(MK + MKA), and MSEC/(MK + MKA), using Euclidean distance and complete linkage. The analysis was considered exploratory and was intended to visualize relative compositional patterns among RYR ingredients rather than to represent a formal compositional-data analysis.

**Figure 2:**
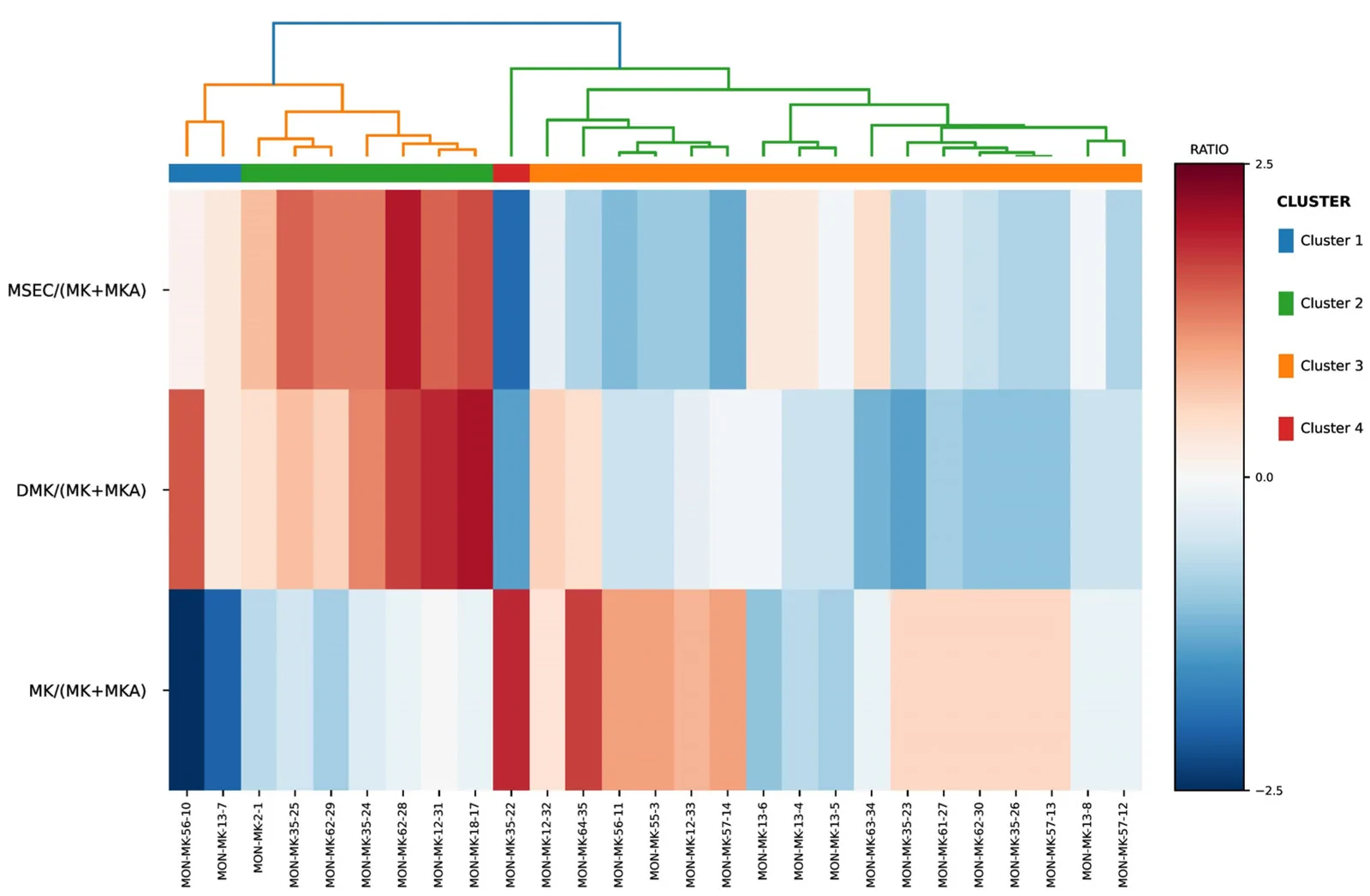
Heatmap illustrating the relative monacolin profiles across the 27 RYR ingredients using MK/(MK + MKA), DMK/(MK + MKA), and MSEC/(MK + MKA). Hierarchical clustering was performed on the unscaled ratios using Euclidean distance and complete linkage. For heatmap visualization only, values were standardized within each variable. Red indicates values above and blue values below the corresponding variable mean. Cluster colors indicate the exploratory groups identified by HCA.

The HCA, visualized as a dendrogram and associated heatmap in Figure 2, was performed using the three non-redundant compositional variables MK/(MK + MKA), DMK/(MK + MKA), and MSEC/(MK + MKA). The analysis revealed distinct clustering patterns among the RYR ingredients. A broad separation was observed between samples characterized by lower MK/(MK + MKA) ratios and relatively higher contributions of DMK and secondary monacolins, and samples characterized by higher MK/(MK + MKA) ratios and generally lower DMK and MSEC values. Within these groups, additional subclusters reflected different combinations of DMK and secondary monacolin abundance. Given the shared denominator among the ratios, these clustering patterns were interpreted as exploratory descriptors of relative compositional variability.

The HCA provided an exploratory overview of compositional similarities among the RYR ingredients and supported the identification of samples displaying distinct monacolin profiles. Six ingredients previously selected for subsequent cytotoxicity testing were mapped onto the clustering solution and are summarized below according to the three non-redundant variables used in the HCA:

MON-MK-35-26 (Cluster 3): MK/(MK + MKA) = 0.91; DMK/(MK + MKA) = 0.02; MSEC/(MK + MKA) = 0.07;

MON-MK-64-35 (Cluster 3): MK/(MK + MKA) = 0.98; DMK/(MK + MKA) = 0.11; MSEC/(MK + MKA) = 0.07;

MON-MK-12-31 (Cluster 2): MK/(MK + MKA) = 0.88; DMK/(MK + MKA) = 0.20; MSEC/(MK + MKA) = 0.20;

MON-MK-56-10 (Cluster 1): MK/(MK + MKA) = 0.72; DMK/(MK + MKA) = 0.18; MSEC/(MK + MKA) = 0.12;

MON-MK-13-6 (Cluster 3): MK/(MK + MKA) = 0.82; DMK/(MK + MKA) = 0.08; MSEC/(MK + MKA) = 0.13;

MON-MK-63-34 (Cluster 3): MK/(MK + MKA) = 0.87; DMK/(MK + MKA) = 0.01; MSEC/(MK + MKA) = 0.14.

**Figure 3:**
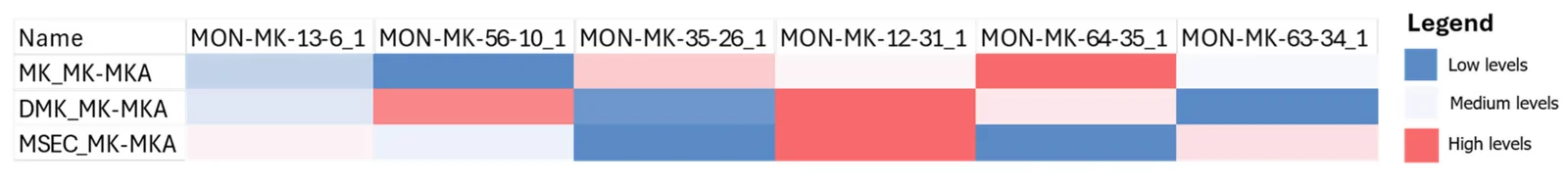
Heatmap illustrating the compositional profiles of the six RYR ingredients selected for subsequent cytotoxicity testing. Each row represents one of the three non-redundant ratios used in the revised analysis—MK/(MK + MKA), DMK/(MK + MKA), and MSEC/(MK + MKA)—while each column corresponds to a specific sample. The heatmap provides an exploratory descriptive visualization of the relative compositional differences among the selected RYR ingredients.

These samples span different combinations of MK, DMK, and secondary monacolin ratios and were therefore retained for biological comparison. The relative compositional profiles of these six selected RYR ingredients are shown in Figure 3. The revised HCA was used as an exploratory visualization of compositional similarity and was not interpreted as providing a formal classification of the samples selected for cytotoxicity testing.

### 3.3. Untargeted Analysis

The analytical workflow was extended beyond monacolin identification to achieve a broader characterization of the chemical composition of RYR ingredients. For this purpose, an exploratory qualitative untargeted metabolomics approach was applied to the six RYR extracts selected for subsequent biological characterization. Each sample was extracted using three ethanol concentrations (30%, 50%, and 75%) to explore solvent-dependent extraction and metabolite variability.

For each extraction condition, compounds identified across the six samples were classified and grouped by chemical class Figure. The full filtered feature lists are reported in the Supplementary Material. Annotation confidence was assigned according to the Metabolomics Standards Initiative (MSI). Only monacolin K (MK) was confirmed with an authentic standard (MSI Level 1). All other compounds were annotated based on accurate mass and MS/MS spectral matching to databases (MSI Level 2) and should be considered putative identifications pending standard confirmation.

**Figure 4:**
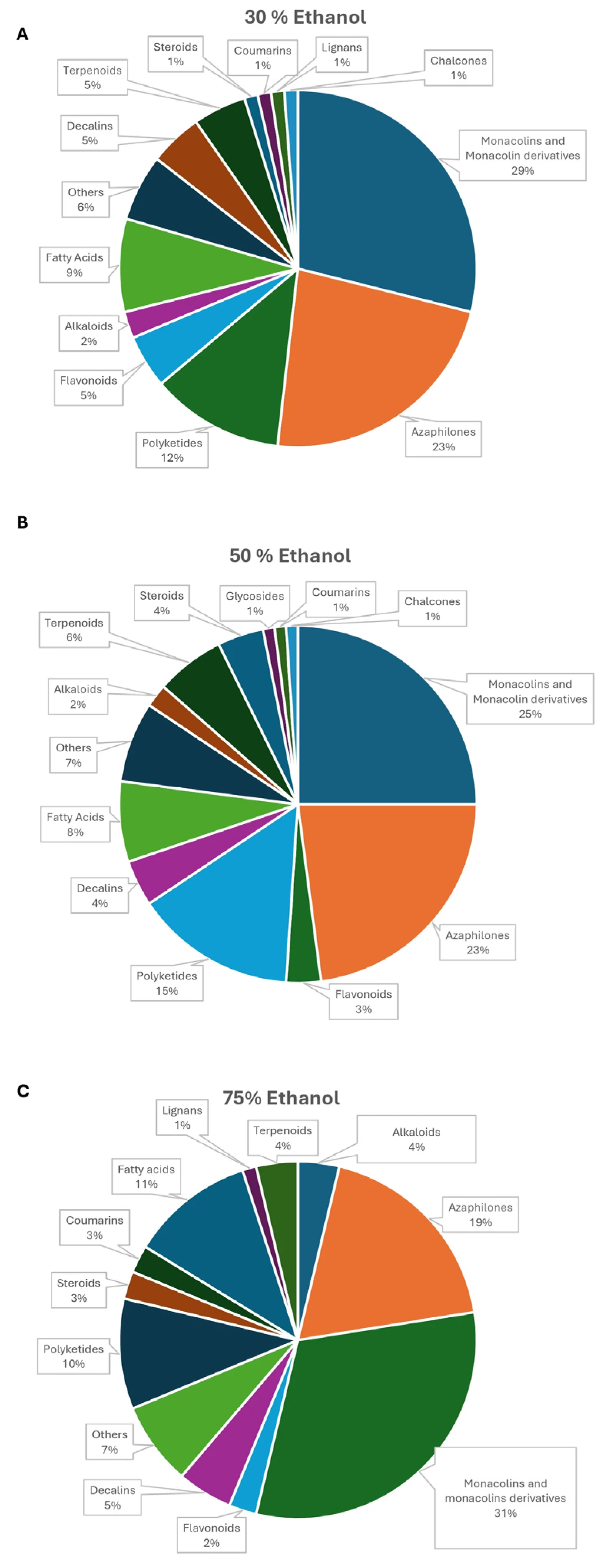
Pie chart showing the overall percentage distribution of metabolite classes identified in (**A**) 30% ethanol extract, (**B**) 50% ethanol extract, and (**C**) 75% ethanol extract. Each sector represents the percentage contribution of a metabolite class to the total number of identified metabolites in the corresponding extract.

With 30% ethanol (Figure 4A), monacolins were the most represented class (29%), followed by azaphilones (23%) and polyketides (12%). A comparable pattern was observed for extracts obtained with 50% ethanol (Figure 4B), where monacolins (25%), azaphilones (23%), and polyketides (15%) remained the predominant groups. Extraction with 75% ethanol (Figure 4C) again yielded monacolins as the predominant class (30%), followed by azaphilones (19%) and fatty acids (11%).

Overall, the same major metabolite classes were detected under all extraction conditions, with differences in their relative proportions. Differences in relative proportions across solvents reflect differential solubility rather than a selective enrichment of MK, which remained consistently detected across all conditions.

A more detailed evaluation of azaphilones, classified as pigments, was conducted to investigate their variety under different solvent conditions. As shown in Table 5, extraction with 50% ethanol enabled the putative annotation of seventeen pigments, compared to fourteen and ten pigments detected using 30% and 75% ethanol, respectively. These data indicate that 50% ethanol provided the broadest qualitative coverage and a greater number of putatively annotated pigments under the tested conditions.

**Figure 5:**
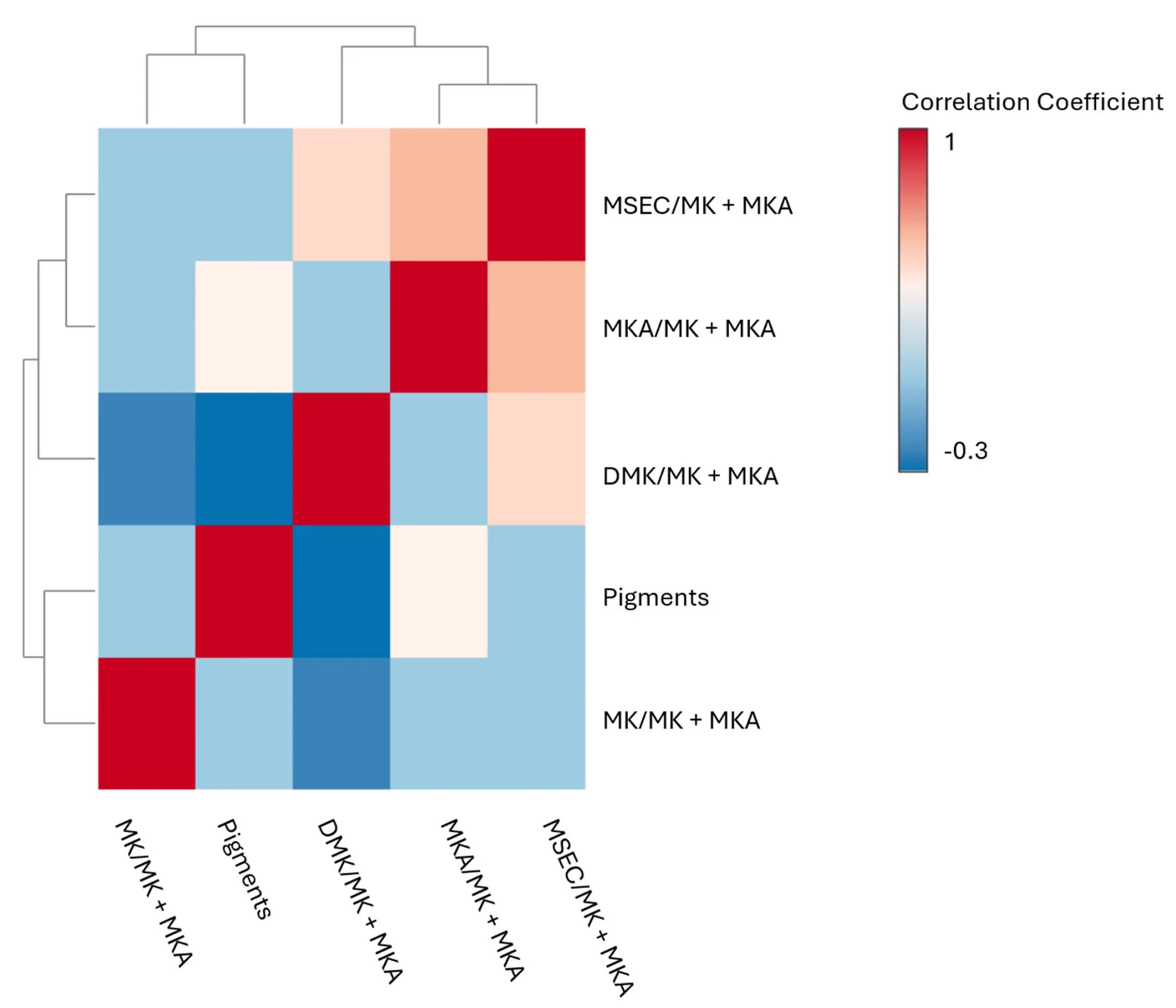
Correlation matrix among the metabolites’ levels (N = 27). Red indicates a direct Pearson correlation coefficient, while blue indicates an inverse correlation. *p*-values from the pairwise Pearson correlation analyses were adjusted using the Benjamini–Hochberg false discovery rate (FDR) procedure; correlations with q < 0.05 were considered statistically significant.

To further elucidate potential relationships among monacolins, pigments, and other metabolites, a Pearson correlation matrix was generated (Figure 5). Five significant correlations were identified. DMK exhibited inverse correlations with both MK and pigments (ρ = −0.30, *p* ≤ 0.02 *; ρ = −0.38, *p* ≤ 0.001 ***). In contrast, secondary monacolins (MSEC) showed direct correlations with both DMK (ρ = 0.45, *p* ≤ 0.0001 ****) and MKA (ρ = 0.58, *p* ≤ 0.002 **). A further direct correlation was observed between MKA and pigments (ρ = 0.36, *p* = 0.002 **). It should be emphasized that these correlation analyses were based on targeted monacolin data and semi-quantitative LC–UV pigment peak intensities acquired at characteristic pigment wavelengths. Therefore, correlation matrices evaluate co-variation patterns, not biosynthetic equivalence, and should be interpreted cautiously, without implying direct metabolic or enzymatic relationships.

### 3.4. Cytotoxicity Results

The cytotoxic potential of six selected RYR extracts was evaluated in HepG2 cells and HSkMC cells to investigate the relationship between monacolin composition and cell viability. Extract concentrations were normalized to the quantified monacolin K content, allowing comparison of cytotoxic responses across RYR extracts differing in the relative abundance of DMK, secondary monacolins, and other matrix constituents under equivalent MK exposure. Treatments revealed distinct cytotoxic trends depending on the relative content of monacolins.

Cell-free interference controls did not show relevant extract-dependent changes in the Alamar Blue fluorescence signal under the experimental conditions employed.

**Figure 6:**
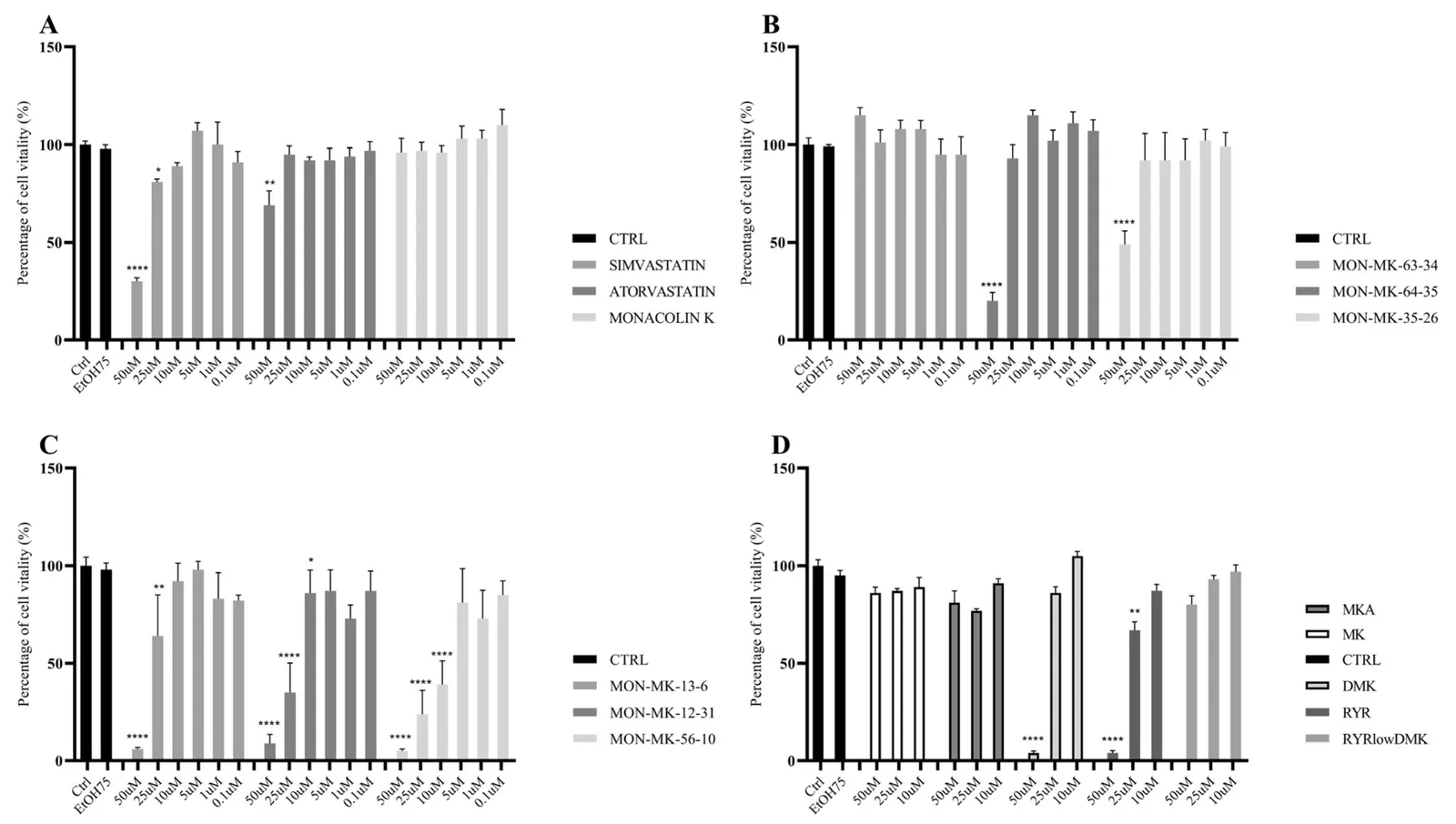
Effect of (**A**) simvastatin, atorvastatin, and MK; (**B**) MON-MK-64-35 and MON-MK-63-34. (**C**) MON-MK-35-26, MON-MK-13-6, MON-MK-12-31 and MON-MK-56-10. (**D**) MK, MKA, DMK, RYR and RYRlowDMK on the viability of HepG2 cells after 24 h of exposure. Cell viability is expressed as a percentage relative to corresponding vehicle control cells. Data represent mean ± SD from three independent biological experiments (n = 3), each performed in technical triplicate. Technical replicates were averaged within each biological experiment before statistical analysis. Statistical significance was assessed by one-way ANOVA, followed by Dunnett’s post hoc test for multiple comparisons between experimental groups and the control group. * ≤0.05; ** ≤0.01; **** ≤0.0001.

For HepG2 cells, results are shown in Figure 6. At the highest dose (50 μM), treatments with MON-MK-64-35 induced a marked cytotoxic effect, with cell viability 80% lower than that of untreated cells. No statistically significant reduction in cell viability was observed at the other tested concentrations. Conversely, MON-MK-63-34 did not produce a statistically significant reduction in cell viability at any tested concentration. MON-MK-35-26 induced a 51% reduction in viability at 50 μM, with negligible effects at lower doses (Figure 6B).

Among the selected extracts, differences in monacolin composition were associated with different reductions in cell viability (Figure 6C). MON-MK-13-6 resulted in decreases in cell viability of 94 and 36% at 50 and 25 μM, respectively. MON-MK-12-31 yielded viability reductions of 91, 65%, and 14% at 50, 25, and 10 μM. Lastly, MON-MK-56-10 exhibited the greatest effect, reducing viability by 95%, 76%, and 61% at 50, 25, and 10 μM, respectively, and slight reductions even at lower doses.

For comparison, standard statins, MK, and the preparatively obtained MKA and DMK fractions were tested (Figure 6A–D). Simvastatin reduced viability by 70% and 19% at the highest doses (50 and 25 µM), with no effect at lower concentrations. Atorvastatin produced a 31% decrease in viability only at 50 μM. Pure MK displayed limited cytotoxicity only at 50 μM.

When the preparatively obtained MKA and DMK fractions were compared with the unfractionated RYR preparation and RYRlowDMK (Figure 6D), DMK and RYR with high DMK induced a 96% reduction in viability at 50 μM, whereas MK, MKA and RYRlowDMK reduced viability by 14, 19 and 20%, respectively. At 25 μM, only RYR with high DMK revealed significant cytotoxicity, and at 10 μM, none of the tested compounds affected cell viability.

Importantly, RYRlowDMK was obtained by removing the late-eluting chromatographic portion containing DMK together with other more strongly retained constituents. Therefore, the lower cytotoxicity observed for RYRlowDMK compared with the corresponding unfractionated RYR preparation cannot be attributed specifically to the reduction of DMK. Rather, this comparison indicates that removal of the late-eluting fraction, including DMK and additional constituents, was associated with a reduction in cytotoxicity.

**Figure 7:**
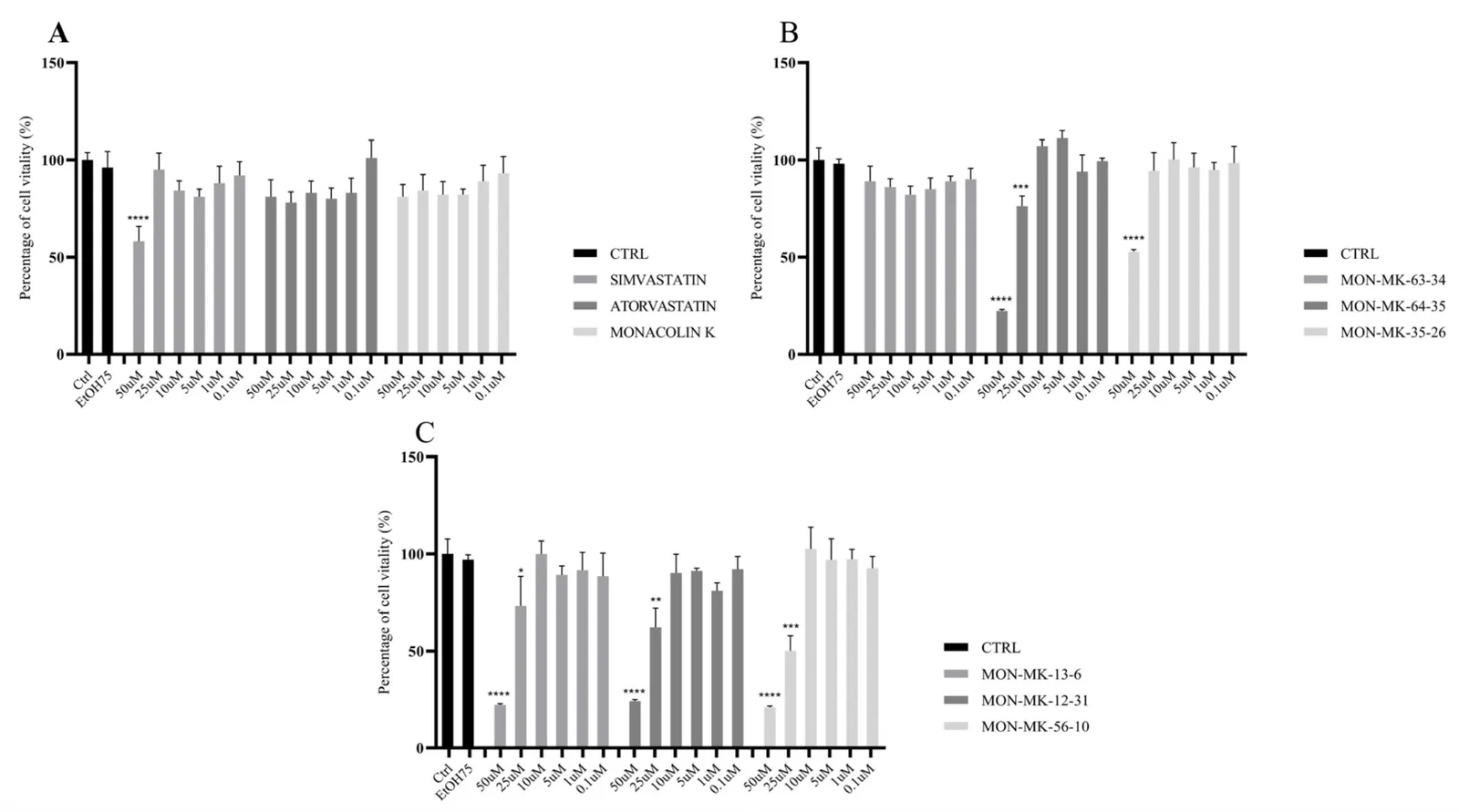
Effect of (**A**) simvastatin, atorvastatin, and MK; (**B**) MON-MK-64-35 and MON-MK-63-34. (**C**) MON-MK-35-26, MON-MK-13-6, MON-MK-12-31 and MON-MK-56-10 on the viability of primary HSkMc cells after 24 h of exposure. Cell viability is expressed as a percentage relative to corresponding vehicle control cells. Data represent mean ± SD from three independent biological experiments (n = 3), each performed in technical triplicate. Technical replicates were averaged within each biological experiment before statistical analysis. Statistical significance was assessed by one-way ANOVA, followed by Dunnett’s post hoc test for multiple comparisons between experimental groups and the control group. * ≤0.05; ** ≤0.01; *** ≤0.001; **** ≤0.0001.

In HSkMC cells, the overall cytotoxic trends mirrored those observed in HepG2 cells, albeit with slightly lower magnitude (Figure 7).

In Figure 7B, MON-MK-63-34 did not significantly affect cell viability at any concentration, whereas MON-MK-64-35 and MON-MK-35-26 resulted in viability losses of 78 and 47% at 50 μM, respectively, with MON-MK-64-35 also producing a 24% decrease at 25 μM.

Among the selected extracts, higher DMK levels were associated with greater reductions in cell viability (Figure 7C). MON-MK-13-6 caused decreases of 78 and 27% at 50 and 25 μM. MON-MK-12-31 produced 76 and 38% reductions at the same concentrations, and MON-MK-56-10 showed the greatest effect, with cell viability 79% and 50% lower than that of untreated cells. Finally, simvastatin reduced viability by 42% at 50 μM, whereas atorvastatin and MK showed a limited 19% decrease only at 50 μM, with no effects at lower concentrations (Figure 7A).

### 3.5. Exploratory Proteomic Analysis

To complement the cytotoxicity experiments, exploratory label-free proteomic profiling was performed to compare protein-abundance patterns among control, RYR-treated (MON-MK-63-34), and simvastatin-treated cells. As described in Section 2.7, three independently cultured and treated biological samples were pooled within each experimental condition before proteomic processing. Consequently, inter-sample biological variability could not be estimated, and the following results should be interpreted as descriptive and hypothesis-generating rather than as confirmatory differential-expression analyses.

#### 3.5.1. HepG2 Cells

Approximately 4246 quantifiable proteins were detected. Exploratory comparison of protein-abundance profiles highlighted sets of proteins showing different abundance patterns among the three experimental conditions.

Since alterations in redox homeostasis are involved in the development and progression of liver diseases [21], the exploratory proteomic analysis focused on protein sets associated with redox homeostasis and DNA conservation and maintenance. In addition, dysregulation of redox homeostasis has been linked to apoptosis and the elimination of cells with excessive DNA damage [22]. Based on the comparison of the proteomic profiles:

- Proteins associated with DNA conservation/maintenance and redox-homeostasis processes were more represented among proteins showing higher abundance in RYR-treated and control cells than in simvastatin-treated cells.
- Conversely, proteins associated with apoptosis-related processes were more represented among proteins showing higher abundance in simvastatin-treated cells than in RYR-treated and control cells.

**Figure 8:**
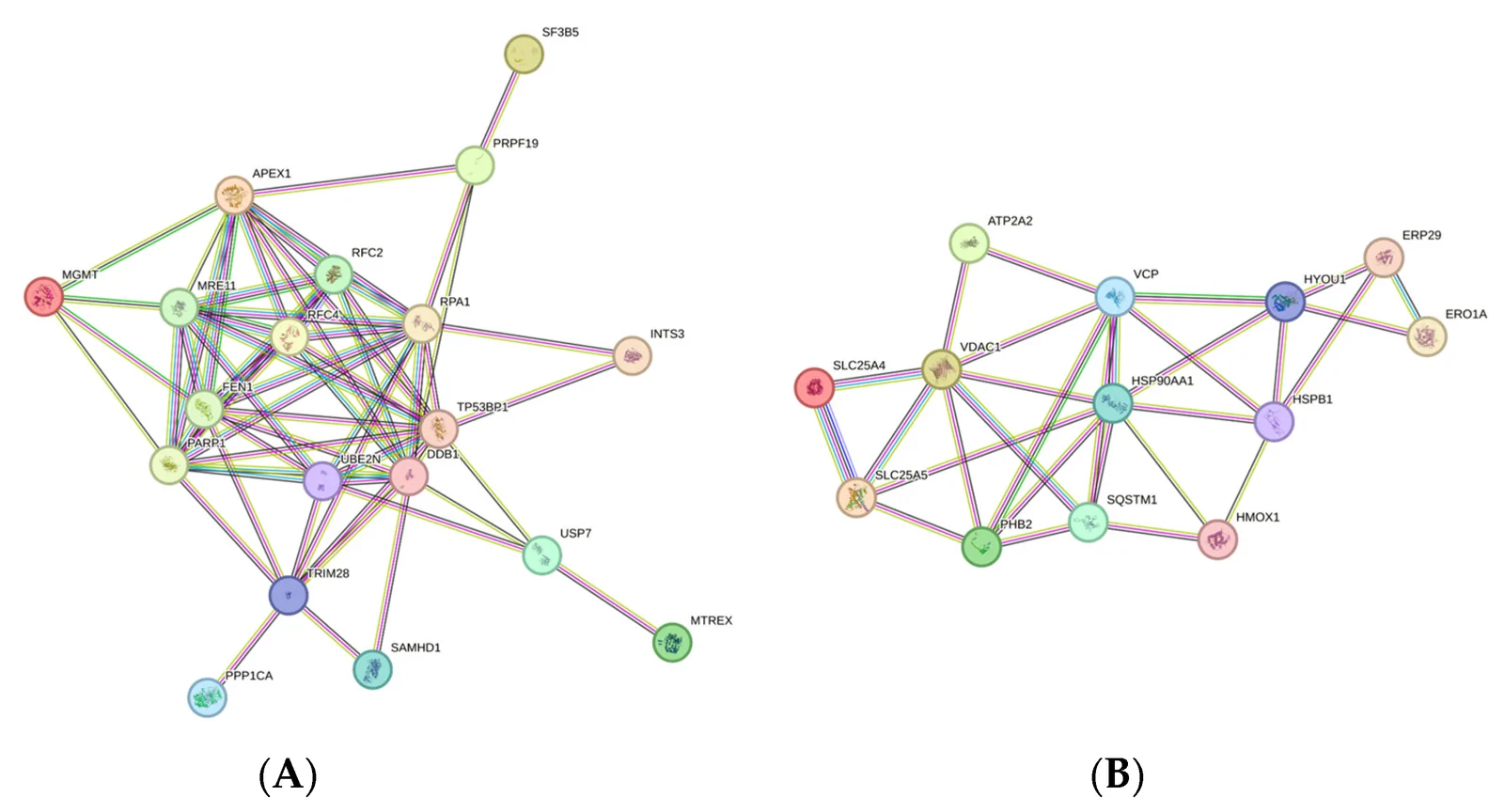
Exploratory protein–protein interaction networks generated using STRING for proteins showing different abundance patterns in (**A**) RYR-treated and control cells and (**B**) simvastatin-treated cells. Each node represents a protein, whereas edges represent known or predicted protein–protein associations. Edge colors indicate the source of evidence supporting each association: light blue, curated databases; pink, experimentally determined interactions; green, gene neighborhood; red, gene fusions; dark blue, gene co-occurrence; light green, text mining; black, co-expression; and indigo, protein homology.

Figure 8A shows the exploratory STRING interaction network generated from proteins displaying higher abundance in RYR-treated and control cells and an opposite abundance pattern following simvastatin treatment. The proteins included in this network are reported in Table 6, together with their database accession numbers, gene names, and protein names.

**Figure 9:**
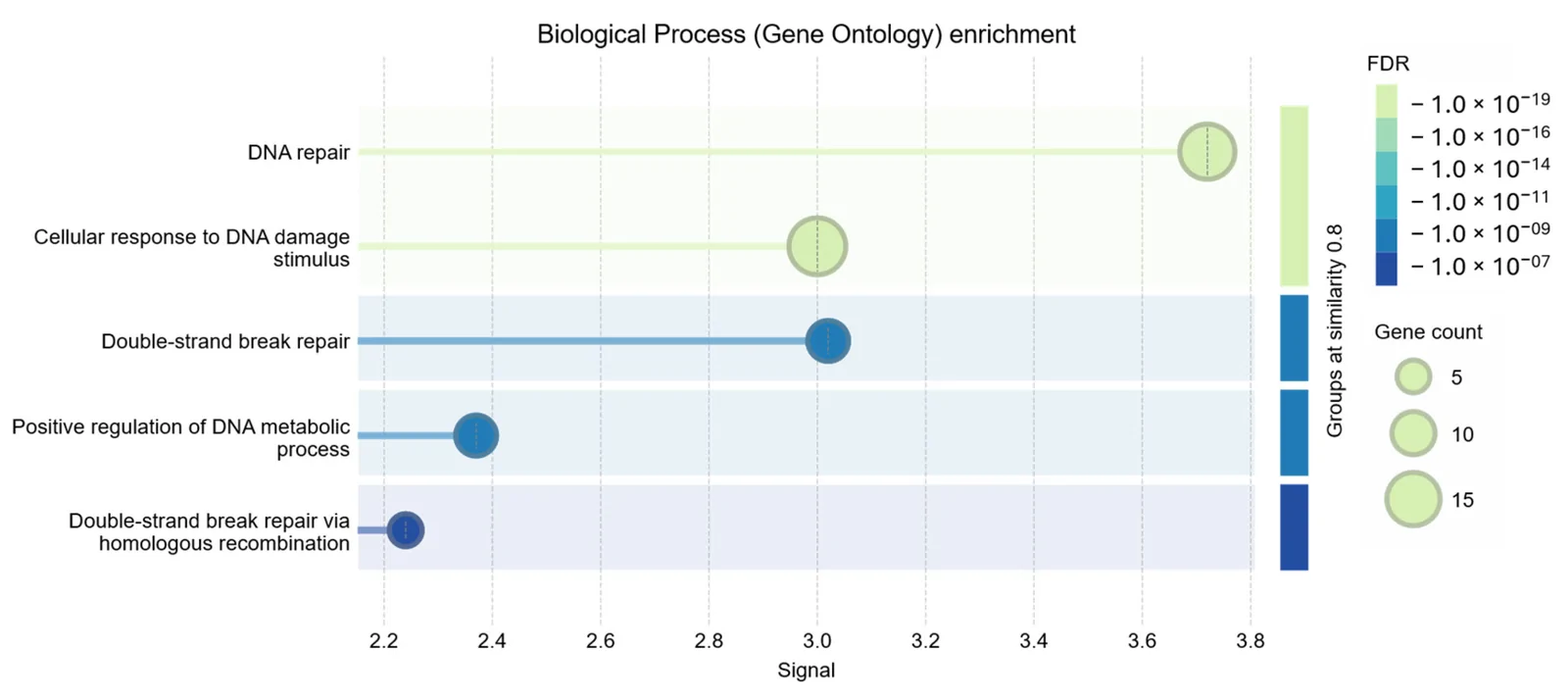
Gene Ontology (GO) enrichment analysis obtained using STRING for the exploratory protein set showing higher abundance in RYR−treated and control cells. The five most represented biological processes are shown. Dot size represents the number of proteins associated with each biological process, whereas color intensity indicates the STRING−reported false discovery rate (FDR). The *x*−axis represents the enrichment signal for each biological process.

To further characterize the functional annotations associated with this exploratory protein set, the functional enrichment tool integrated in STRING was applied (Figure 9). The enrichment analysis highlighted “DNA repair” as the most represented biological process, followed by “cellular response to DNA damage stimulus” and other processes related to DNA metabolic homeostasis. These results indicate over-representation of these functional annotations within the selected protein set and should not be interpreted as evidence of pathway activation.

Among the 19 proteins showing higher abundance in RYR-treated and control cells, PARP1 and APEX1 have been associated with redox-homeostasis-related functions, whereas several of the remaining proteins are functionally annotated to processes related to DNA conservation, DNA repair, and regulation of DNA metabolic processes.

The exploratory STRING interaction network generated from proteins showing higher abundance following simvastatin treatment is shown in Figure 8B. The proteins included in this network are listed in Table 7. Functional annotation of this protein set indicated associations with processes related to macroautophagy, mitochondrial function, endoplasmic reticulum stress, and apoptosis-related pathways. These associations describe the functional characteristics of the selected protein set and should not be interpreted as evidence of activation of these pathways.

**Figure 10:**
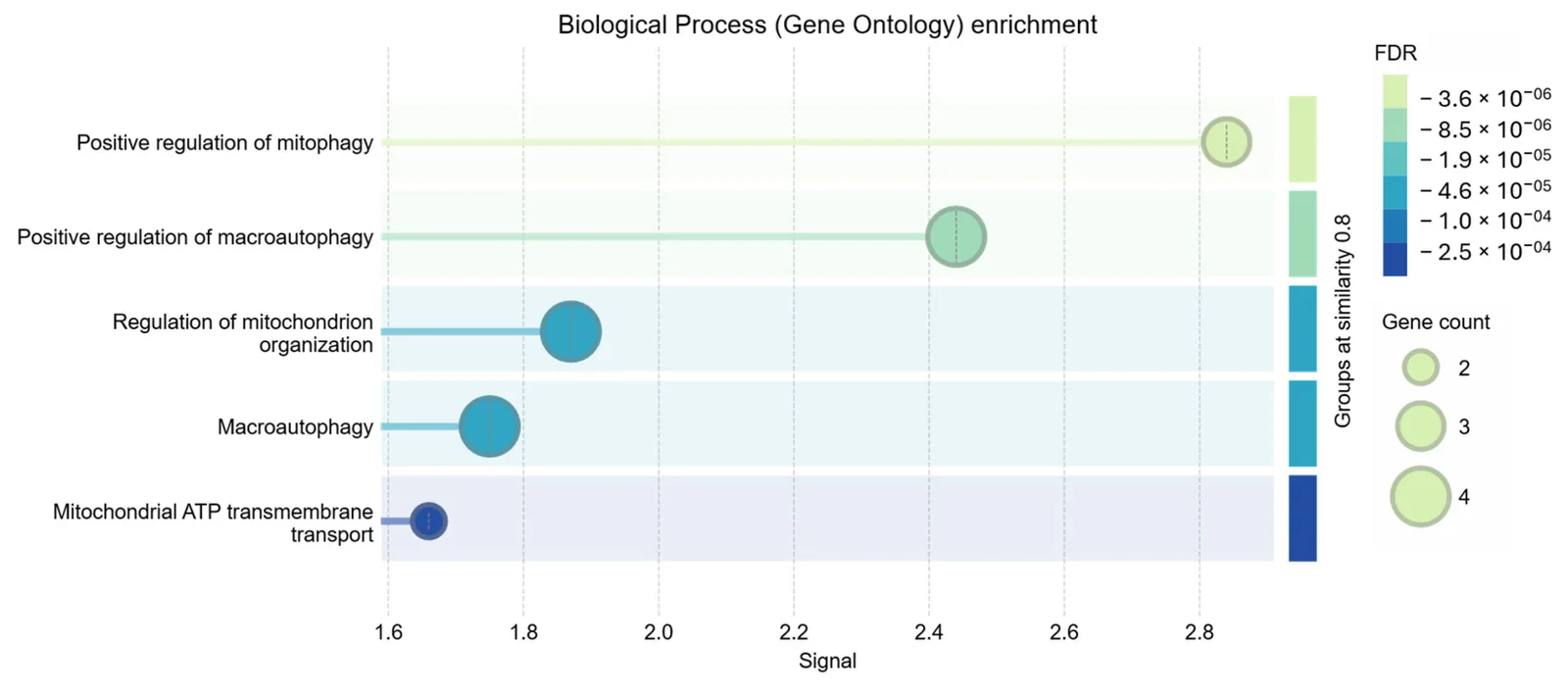
Gene Ontology (GO) enrichment analysis obtained using STRING for the exploratory protein set showing higher abundance following simvastatin treatment. The five most represented biological processes are shown. Dot size represents the number of proteins associated with each biological process, whereas color intensity indicates the STRING-reported false discovery rate (FDR). The *x*-axis represents the enrichment signal for each biological process.

As previously done, functional enrichment was used to obtain the most enriched biological process (Figure 10). The five most intense signals of the biological process enrichment were all linked to mitochondrial autophagy and degradation.

Exploratory functional enrichment of the selected protein sets suggested distinct functional patterns between the two treatments. Proteins showing higher abundance in the simvastatin condition were associated with over-represented terms related to cellular stress and mitochondrial/autophagic processes, whereas the RYR-associated protein set showed over-representation of different cellular processes. These observations are hypothesis-generating and require validation in experiments in which independent biological samples are retained separately throughout the proteomic workflow.

In addition to the exploratory functional and network analyses, selected proteins previously associated with hepatic homeostasis and cellular stress responses were examined. These included aspartate aminotransferase (AST), glutathione S-transferase (GST), and superoxide dismutase (SOD), which have been reported in the literature in relation to hepatic injury, detoxification, and oxidative-stress responses, respectively [23,24,25]. In the present dataset, these proteins showed abundance patterns in RYR-treated cells that were more similar to those observed in control cells than to those observed in simvastatin-treated cells. Because the three independently cultured and treated biological samples within each condition were pooled prior to proteomic analysis, inter-sample biological variability could not be estimated. These observations should therefore be considered descriptive and hypothesis-generating and should not be interpreted as evidence of preserved hepatic homeostasis or reduced cellular stress.

#### 3.5.2. HSkMC Cells

Approximately 2918 quantifiable proteins were detected. Exploratory comparison highlighted proteins showing different abundance patterns among control, RYR-treated, and simvastatin-treated conditions. The proteomic analysis focused on proteins previously associated with muscle damage and muscle-related cellular processes in the literature [26].

HSkMC cells treated with RYR showed a set of proteins with higher abundance displaying a pattern similar to that observed in control cells and different from that observed following simvastatin treatment. These proteins have previously been associated with ribosomal function, extracellular matrix organization, cytoskeletal regulation, and calcium-binding processes (Table 8):

- Ribosomal proteins (RPS26, RPL10): these proteins have been associated with the maintenance of intracellular protein homeostasis and muscle protein turnover [27], processes relevant to tissue remodelling and repair.
- Collagen isoforms (COL1A1, COL1A2): changes in the abundance of these proteins have been associated with extracellular matrix organization and processes contributing to force transmission between muscle and connective tissues [28].
- Cytoskeletal and calcium-binding proteins (Gelsolin, Sorcin, SDF4): these proteins have been associated with actin-cytoskeleton regulation and intracellular calcium-related processes relevant to muscle structural organization and contraction [29,30].

Under the STRING analysis parameters applied, this protein set did not yield a statistically enriched protein–protein interaction network.

In contrast, simvastatin-treated skeletal muscle cells showed higher abundance of a set of eight proteins displaying a different pattern compared with control and RYR-treated cells (Table 9). Based on previously reported functions, these proteins have been associated with several cellular processes relevant to skeletal muscle:

- Proteins associated with muscle atrophy and protein catabolism: some of the proteins showing higher abundance have previously been implicated in pathways related to protein degradation and muscle-remodeling processes [31].
- Proteins associated with extracellular matrix remodeling and fibrosis: metalloproteinase inhibitor 1 (TIMP1) has been implicated in extracellular tissue remodeling and fibrotic processes [32].
- Proteins associated with endoplasmic reticulum (ER) and cellular stress: some of the identified proteins have previously been associated with protein-folding homeostasis, ER stress, and cellular responses to metabolic stress [33]. Prolonged ER stress has been linked in the literature to apoptotic signaling [33].

Exploratory STRING network analysis highlighted functional associations among some of these proteins. In particular, DNM2, HYOU1, and PHB have been linked in the literature to mitochondrial function and cellular stress-related processes, whereas PLXNB2 and RRAS have been associated with pathways relevant to muscle remodeling. These associations provide biological context for the observed protein-abundance patterns but should be considered hypothesis-generating. Because the three independently cultured and treated biological samples within each experimental condition were pooled prior to proteomic analysis, inter-sample biological variability could not be estimated. These observations therefore require validation in experiments in which independent biological samples are retained separately throughout the proteomic workflow.

## 4. Discussion

In 2018, EFSA raised concerns regarding red yeast rice (RYR) products, highlighting that monacolin K (structurally identical to lovastatin) shares the adverse effects of statins and, together with other monacolins of unestablished safety, may expose consumers to health risks [6]. EFSA concluded that safety concerns exist at intakes of 3 mg/day or higher and, in its subsequent 2025 opinion, stated that it was not possible to establish a safe daily intake of MK from RYR products even at lower doses [12]. At the European Union level, Commission Regulation (EU) 2022/860 [34] requires the recommended daily portion to provide less than 3 mg of monacolins and establishes mandatory labelling requirements. However, it does not define comprehensive compositional standards for individual monacolins or other secondary metabolites, and risk-based official controls do not involve systematic pre-market or batch-by-batch analysis of every product.

Consequently, scientific uncertainty remains regarding both the safety of monacolin intakes below the regulatory threshold and the compositional variability of commercially available RYR products. Previous studies have reported substantial variability in total monacolins, MK, and monacolin K acid (MKA), which may be relevant to product tolerability and safety [35]. Within this context, the present study aimed to provide experimental evidence supporting a more comprehensive approach to RYR characterization, combining chemical profiling with biological evaluation, in order to identify compositional features potentially relevant for safety monitoring.

A representative group of twenty-seven commercial RYR ingredients was analyzed to provide a snapshot of the current market. Untargeted metabolomic analysis revealed a complex chemical composition extending beyond monacolins, including azaphilones, polyketides, and lipid compounds. None of the samples contained synthetic statins, and all complied with regulatory limits for citrinin. However, substantial qualitative and quantitative differences were observed among products, indicating pronounced inter-producer variability, as confirmed already in some precedent monitoring where it was reported that botanicals based on RYR on the market have a significantly varied profile [36]. The untargeted approach was not intended for definitive structural elucidation of all detected compounds but rather to provide a comprehensive overview of metabolite classes and compositional variability across products in order to evaluate whether the possible adverse events stem only from single specific metabolites or from the whole matrix.

Correlation analyses highlighted non-random associations between specific monacolins and accessory metabolites. In particular, MKA was positively associated with azaphilone pigments, whereas DMK showed an inverse correlation with pigment abundance. These findings suggest that the metabolic dynamics of the product influence not only the amount of monacolins but also that of accessory compounds, with possible consequences on the risk/benefit balance. It is important to emphasize that these correlations are based on semi-quantitative peak intensities and reflect co-variation across samples rather than biosynthetic or causal relationships. Although azaphilone pigments are known to possess biological activities, including antimicrobial and cytotoxic effects [37,38], the present data indicate that pigment abundance alone does not directly explain cytotoxic outcomes. Rather, pigment levels appear to reflect underlying metabolic patterns associated with specific monacolin distributions.

A novel and relevant aspect emerging from this study concerns the potential role of dehydromonacolin K (DMK). Although already reported as a secondary metabolite [7], DMK variability is substantial among commercial ingredients and, most importantly, associated with increased cytotoxicity in cell models. In particular, in vitro experiments demonstrated that RYR samples with high DMK content induced significant reductions in cell viability in human hepatocellular carcinoma cells and human primary skeletal muscle cells, representing the most affected tissues from statin toxicity. In contrast, MK and MKA, tested individually at the same concentrations, did not induce comparable cytotoxic responses under the same experimental conditions. Accordingly, MK and MKA alone were not sufficient to explain the variability in cellular responses observed among RYR extracts. The additional comparison between the unfractionated RYR preparation and RYRlowDMK should be interpreted with particular caution. RYRlowDMK was obtained by removing the late-eluting chromatographic portion containing DMK together with other more strongly retained constituents and therefore does not represent a selectively DMK-depleted preparation. Although RYRlowDMK showed lower cytotoxicity than the corresponding unfractionated RYR preparation, this difference cannot be attributed specifically to DMK and may reflect the combined removal of DMK and other late-eluting components.

RYR samples characterized by low DMK levels, high MK content, and high pigment abundance did not induce significant cytotoxicity, whereas extracts with elevated DMK/MK + MKA ratios and low pigment levels showed pronounced toxic effects. These observations suggest that cytotoxicity associated with certain RYR products is not exclusively attributable to MK but rather reflects the combined contribution of specific monacolin profiles and accessory metabolites, with DMK emerging as a candidate compositional marker. However, given the observational and in vitro nature of the study, the role of DMK should be interpreted as strongly associated with cytotoxicity rather than conclusively causal. Previous in vitro studies have reported cytotoxic activity for dehydromonacolin [10], while clinical observations indicate that adverse reactions to RYR products cannot be explained solely by MK content [6,11].

The comparison with synthetic statins further contextualized these findings. As expected, simvastatin exhibited greater cytotoxicity than atorvastatin, in agreement with previous reports [39,40]. Importantly, only a subset of RYR extracts induced toxic effects comparable to those of statins, and this occurred in a composition-dependent manner. This supports the hypothesis that the overall biological effect of RYR results from the interaction between multiple monacolins and secondary metabolites, rather than from MK alone, in line with earlier hypotheses proposed for azaphilone-containing preparations [37,38]. Nevertheless, the marked reductions in cell viability observed for some RYR extracts indicate that in vitro cellular responses may vary according to product composition and were associated, in the selected samples, with DMK levels and other metabolites.

Exploratory proteomic profiling provided complementary, hypothesis-generating information on protein-abundance patterns following RYR and simvastatin exposure. In both HepG2 and HSkMC cells, the selected low-DMK RYR ingredient and simvastatin were associated with different protein-abundance and functional-enrichment patterns. Proteins showing higher abundance following simvastatin treatment were associated with cellular stress, mitochondrial function, autophagy, and related processes, whereas different functional patterns were observed following RYR treatment. However, although three independent biological cell cultures were prepared and treated separately for each experimental condition, these samples were pooled prior to proteomic analysis. Consequently, inter-sample biological variability could not be estimated, and the observed protein-abundance and enrichment patterns cannot be interpreted as statistically validated biological responses or mechanistic evidence. These findings should therefore be considered exploratory and hypothesis-generating and require confirmation in experiments in which independent biological samples are retained separately throughout the proteomic workflow.

Several limitations should be acknowledged. Regarding sample representativeness, the limited sample size, lack of systematic geographical and batch stratification, and absence of multiple batches per material limit the generalizability of the findings and preclude assessment of batch-to-batch variability. Moreover, untargeted analysis was exploratory and qualitative and did not provide absolute quantification for all detected metabolites, and causal relationships between individual compounds and biological effects cannot be definitively established. The study is based on in vitro models and correlation analyses and therefore does not allow direct extrapolation to clinical risk. The concentration range was selected to generate concentration-response profiles and to enable comparative assessment under standardized in vitro conditions; it was not intended to reproduce or predict concentrations achievable in human plasma or tissues. A major limitation of the proteomic component is that the three independently cultured and treated biological samples within each experimental condition were pooled prior to proteomic analysis. Although the resulting pooled samples incorporated material from three independent biological cultures, pooling prevented estimation of inter-sample biological variability. The three subsequent technical replicates therefore reflected technical reproducibility of sample preparation and LC–MS/MS analysis rather than independent biological variation. Consequently, protein-abundance comparisons and downstream network and functional-enrichment analyses should be regarded as exploratory and hypothesis-generating rather than as confirmatory evidence of differential protein expression or pathway modulation.

Overall, the present findings support the need for improved regulation of RYR-based products, including standardization of monacolin profiles and monitoring of potentially relevant secondary metabolites such as DMK. The pronounced inter-producer variability observed highlights the importance of post-production controls, as already emphasized by EFSA. The observed variability suggests that quality-control strategies based predominantly on MK or total monacolin content may not fully capture the chemical and biological heterogeneity of commercial RYR ingredients. However, the present in vitro data do not demonstrate that specific compositional profiles cause statin-like adverse effects in consumers. Rather, they identify compositional features and candidate markers that warrant further targeted toxicological and clinical investigation.

In addition, the RYRlowDMK preparation was obtained by removing a late-eluting chromatographic fraction containing DMK together with other retained constituents. Therefore, the comparison between the unfractionated RYR preparation and RYRlowDMK cannot isolate the specific contribution of DMK to the observed cytotoxic response.

An additional limitation is that cell viability was assessed using the Alamar Blue assay only. Although cell-free interference controls did not indicate relevant extract-dependent changes in fluorescence, confirmation using an independent orthogonal viability or cytotoxicity assay was not performed.

## 5. Conclusions

This study provides a comprehensive chemical and biological characterization of twenty-seven commercial red yeast rice (RYR) ingredients, highlighting substantial variability in monacolin profiles and secondary metabolites across products. Beyond MK and MKA, secondary monacolins, particularly DMK, emerged as candidate compositional markers. The integration of chemical profiling and in vitro cytotoxicity assays indicated that variability in DMK content was associated with differential cellular responses, whereas MK and MKA alone were not sufficient to explain the observed effects. Exploratory proteomic profiling provided complementary hypothesis-generating information on protein-abundance patterns following RYR and simvastatin exposure but was not used as confirmatory evidence of the association between DMK content and cellular responses. These findings support the relevance of considering the overall metabolic profile of RYR supplements, rather than focusing exclusively on MK content, when evaluating product characteristics. Although the results are based on in vitro models and do not allow direct extrapolation to clinical risk, they underscore the importance of comprehensive compositional monitoring of RYR supplements. Overall, the data support the need for improved quality control strategies addressing both monacolin composition and secondary metabolites, in line with current EFSA concerns regarding the safety and standardization of RYR-based products.

## Figures and Tables

**Table 1:** Samples analyzed and relative description.

Identifier	Description
MON-MK-35-22	Red yeast rice (seed) dry extract ≥ 3% monacolins
MON-MK-35-23	Red yeast rice (seed) dry extract ≥ 5% monacolins
MON-MK-35-24	Red yeast rice (seed) dry extract ≥ 1.5% monacolins
MON-MK-35-25	Red yeast rice (seed) dry extract ≥ 3% monacolins
MON-MK-35-26	Red yeast rice (seed) dry extract ≥ 5% monacolins
MON-MK-61-27	Red yeast rice extract 5% monacolins
MON-MK-62-28	Red yeast rice extract ≥ 1.5% monacolins
MON-MK-62-29	Red yeast rice extract ≥ 3% monacolins
MON-MK-62-30	Red yeast rice extract ≥ 5% monacolins
MON-MK-12-31	Red yeast rice dry extract ≥ 1.5%
MON-MK-12-32	Red yeast rice dry extract ≥ 3%
MON-MK-12-33	Red yeast rice dry extract ≥ 5%
MON-MK-56-11	Red yeast rice extract 3% monacolins
MON-MK-56-10	Red yeast rice extract 0.4% monacolins
MON-MK-63-34	Red yeast rice (no specification)
MON-MK-64-35	Soft red yeast rice extract 2%
MON-MK-2-1	Red yeast rice extract ≥ 3% monacolin K
MON-MK-55-3	Red yeast rice (no specification)
MON-MK-13-4	Red yeast rice dry extract (no specification)
MON-MK-13-5	Red yeast rice dry extract (no specification)
MON-MK-13-6	Red yeast rice dry extract (no specification)
MON-MK-13-7	Red yeast rice dry extract (no specification)
MON-MK-13-8	Red yeast rice dry extract (no specification)
MON-MK-57-12	Red yeast rice 4.8% monacolin tot.
MON-MK-57-13	Red yeast rice extract ≥ 5%
MON-MK-57-14	red yeast rice 5%
MON-MK-18-17	Red yeast rice dry extract ≥ 1.5%

**Table 2:** List of monacolins monitored by LC-HRMS.

Compounds	Formula	*m*/*z* (M+H)^+^	RT (min)
Monacolin K (MK)	C24H36O5	405.26355	20.29
Acidic monacolin K (MKA)	C24H38O6	423.27412	17.28
Dehydromonacolin K (DMK)	C24H34O4	387.25299	24.59
Monacolin L	C19H28O3	305.21112	18.13
Compactin	C23H34O5	391.24790	18.59
Monacolin J	C19H28O4	321.20604	9.80
Acidic monacolin L (MLA)	C19H30O4	323.22169	14.90
Monacolin X	C24H34O6	419.24282	15.27
Monacolin M	C23H34O6	407.24282	23.50

**Table 3:** Percentage value of identified monacolins in the samples analyzed, total percentage measured, and total percentage declared. MK concentrations are absolute, based on the external calibration curve; MKA, DMK, and MSEC (intended as sum of other monacolins reported in Table 2) are reported as MK equivalents. Results are reported as mean ± SD as % *w*/*w*. * represents ingredients below their declared minimum specifications.

SAMPLES ID	MK (%)	MKA (%)	DMK (%)	MSEC (%)	TOT (%)	TOT (%) DECLARED
MON-MK-35-22	3.43 ± 0.8	0.03 ± 0.01	0.01 ± 0.01	0.00 ± 0.00	3.47	3
MON-MK-35-23 *	3.81 ± 0.5	0.38 ± 0.03	0.01 ± 0.01	0.28 ± 0.05	4.48	5
MON-MK-35-24	1.21 ± 0.2	0.19 ± 0.01	0.23 ± 0.01	0.27 ± 0.06	1.89	1.5
MON-MK-35-25	2.04 ± 0.6	0.37 ± 0.02	0.30 ± 0.02	0.48 ± 0.10	3.19	3
MON-MK-35-26	4.15 ± 0.5	0.42 ± 0.01	0.11 ± 0.01	0.32 ± 0.06	4.99	5
MON-MK-61-27	4.03 ± 0.4	0.42 ± 0.02	0.15 ± 0.01	0.38 ± 0.08	4.99	ND
MON-MK-62-28	0.97 ± 0.04	0.15 ± 0.02	0.21 ± 0.02	0.25 ± 0.05	1.58	1.5
MON-MK-62-29 *	1.64 ± 0.3	0.33 ± 0.02	0.23 ± 0.02	0.38 ± 0.08	2.58	3
MON-MK-62-30 *	3.60 ± 0.3	0.37 ± 0.02	0.09 ± 0.01	0.32 ± 0.06	4.37	5
MON-MK-12-31	1.08 ± 0.1	0.15 ± 0.01	0.25 ± 0.01	0.25 ± 0.05	1.73	1.5
MON-MK-12-32 *	1.39 ± 0.2	0.16 ± 0.02	0.18 ± 0.02	0.15 ± 0.03	1.88	3
MON-MK-12-33 *	2.97 ± 0.3	0.22 ± 0.02	0.22 ± 0.02	0.20 ± 0.03	3.61	5
MON-MK-56-11	2.55 ± 0.1	0.16 ± 0.02	0.15 ± 0.01	0.15 ± 0.03	3.00	3
MON-MK-56-10	0.25 ± 0.01	0.10 ± 0.01	0.06 ± 0.01	0.04 ± 0.01	0.45	0.4
MON-MK-63-34	5.92 ± 0.3	0.91 ± 0.09	0.09 ± 0.01	0.93 ± 0.10	7.84	ND
MON-MK-64-35	2.68 ± 0.04	0.07 ± 0.01	0.31 ± 0.01	0.19 ± 0.03	3.24	2
MON-MK-2-1	2.16 ± 0.4	0.41 ± 0.02	0.29 ± 0.02	0.42 ± 0.03	3.28	3
MON-MK-55-3	4.08 ± 0.4	0.24 ± 0.02	0.22 ± 0.02	0.24 ± 0.03	4.79	ND
MON-MK-13-4	1.58 ± 0.5	0.31 ± 0.07	0.10 ± 0.02	0.25 ± 0.05	2.24	ND
MON-MK-13-5	1.62 ± 0.3	0.34 ± 0.01	0.10 ± 0.01	0.22 ± 0.04	2.28	ND
MON-MK-13-6	1.35 ± 0.4	0.30 ± 0.01	0.13 ± 0.01	0.21 ± 0.07	2.00	ND
MON-MK-13-7	1.40 ± 0.2	0.45 ± 0.02	0.18 ± 0.03	0.24 ± 0.08	2.26	ND
MON-MK-13-8	1.98 ± 0.3	0.30 ± 0.04	0.12 ± 0.02	0.25 ± 0.06	2.65	ND
MON-MK-57-12 *	2.53 ± 0.4	0.46 ± 0.04	0.14 ± 0.01	0.25 ± 0.05	3.38	5
MON-MK-57-13 *	3.59 ± 0.5	0.37 ± 0.02	0.09 ± 0.01	0.28 ± 0.04	4.33	5
MON-MK-57-14 *	3.41 ± 0.6	0.20 ± 0.01	0.27 ± 0.03	0.15 ± 0.03	4.04	5
MON-MK-18-17	0.94 ± 0.1	0.15 ± 0.01	0.23 ± 0.04	0.23 ± 0.02	1.55	1.5

**Table 4:** Ratios of monacolins in samples analyzed expressed as single monacolin/MK + MKA.

SAMPLES ID	MK/MK + MKA	MKA/MK + MKA	DMK/MK + MKA	MSEC/MK + MKA
MON-MK-35-22	0.99	0.01	0.00	0.00
MON-MK-35-23	0.91	0.09	0.00	0.07
MON-MK-35-24	0.86	0.14	0.16	0.19
MON-MK-35-25	0.85	0.15	0.13	0.20
MON-MK-35-26	0.91	0.09	0.02	0.07
MON-MK-61-27	0.91	0.09	0.03	0.09
MON-MK-62-28	0.87	0.13	0.19	0.23
MON-MK-62-29	0.83	0.17	0.12	0.19
MON-MK-62-30	0.91	0.09	0.02	0.08
MON-MK-12-31	0.88	0.12	0.20	0.20
MON-MK-12-32	0.90	0.10	0.12	0.10
MON-MK-12-33	0.93	0.07	0.07	0.06
MON-MK-56-11	0.94	0.06	0.05	0.05
MON-MK-56-10	0.72	0.28	0.18	0.12
MON-MK-63-34	0.87	0.13	0.01	0.14
MON-MK-64-35	0.98	0.02	0.11	0.07
MON-MK-2-1	0.84	0.16	0.11	0.16
MON-MK-55-3	0.94	0.06	0.05	0.06
MON-MK-13-4	0.84	0.16	0.05	0.13
MON-MK-13-5	0.83	0.17	0.05	0.11
MON-MK-13-6	0.82	0.18	0.08	0.13
MON-MK-13-7	0.76	0.24	0.10	0.13
MON-MK-13-8	0.87	0.13	0.05	0.11
MON-MK-57-12	0.87	0.14	0.05	0.07
MON-MK-57-13	0.91	0.09	0.02	0.07
MON-MK-57-14	0.94	0.06	0.08	0.04
MON-MK-18-17	0.87	0.13	0.21	0.21

**Table 5:** Qualitative distribution of azaphilones/pigments found in RYR ingredients with different extraction solvents (30%, 50% and 75% ethanol); Y—presence, N—absence.

Pigments	Ethanol 30%	Ethanol 50%	Ethanol 75%
Rubropunctamine	Y	Y	Y
Monapurone A	Y	Y	Y
Monascorubramine	Y	Y	Y
9-Hexanoyl-3-(2-hydroxypropyl)-6a-methyl-9,9a-dihydro-6H-furo [2,3-h]isochromene-6,8(6aH)-dione	Y	Y	Y
Monascin	Y	Y	Y
Monapilosusazaphilone	N	Y	Y
Monascopyridine A	Y	Y	Y
Monapurfluore A	Y	Y	Y
Monascopyridine D	N	Y	N
Xanthomonasin B	Y	Y	N
Monasfluore A	Y	Y	Y
Xanthomonasin A	Y	Y	N
Monascopyridine E	Y	Y	Y
Ankaflavin	Y	Y	N
Monascopyridine C	N	Y	N
Monasfluore B	Y	Y	N
Monascopyridine B	Y	Y	N

**Table 6:** Proteins included in the exploratory STRING interaction network showing higher abundance in RYR-treated and control cells.

Accession	Gene Names	Protein Names
P16455	MGMT	Methylated-DNA-protein-cysteine methyltransferase
Q5TZP7	APEX1	DNA repair nuclease/redox regulator APEX1
Q9BWJ5	SF3B5	Splicing factor 3B subunit 5
Q9UMS4	PRPF19	Pre-mRNA-processing factor 19
P42285	MTREX	Exosome RNA helicase MTR4
Q93009	USP7	Ubiquitin carboxyl-terminal hydrolase 7
Q9Y3Z3	SAMHD1	Deoxynucleoside triphosphate triphosphohydrolase SAMHD1
P62136	PPP1CA	Serine/threonine-protein phosphatase PP1-alpha catalytic subunit
Q13263	TRIM28	Transcription intermediary factor 1-beta
V9HW41	UBE2N	Ubiquitin-conjugating enzyme E2 N
Q16531	DDB1	DNA damage-binding protein 1
A6NNK5	TP53BP1	TP53-binding protein 1
Q68E01	INTS3	Integrator complex subunit 3
P27694	RPA1	Replication protein A 70 kDa DNA-binding subunit
P35249	RFC4	Replication factor C subunit 4
P09874	PARP1	Poly [ADP-ribose] polymerase 1
Q6FHX6	FEN1	Flap endonuclease 1
P49959	MRE11	Double-strand break repair protein MRE11
Q75MT5	RFC2	Replication factor C subunit 2

**Table 7:** Proteins included in the exploratory STRING interaction network showing higher abundance following simvastatin treatment.

Accession	Gene Names	Protein Names
A0A0S2Z3H3	SLC25A4	ADP/ATP translocase 1
P05141	SLC25A5	ADP/ATP translocase 2
A0A1L1UHR1	VDAC1	Non-selective voltage-gated ion channel VDAC1
A0A0S2Z3L2	ATP2A2	Sarcoplasmic/endoplasmic reticulum calcium ATPase 2
Q99623	PHB2	Prohibitin-2
Q13501	SQSTM1	Sequestosome-1
K9JA46	HSP90AA1	Heat shock protein HSP 90-alpha
P55072	VCP	Transitional endoplasmic reticulum ATPase
A0A384P5T6	HYOU1	Hypoxia up-regulated protein 1
V9HW43	HSPB1	Heat shock protein beta-1
Q6FH11	HMOX1	Heme oxygenase 1
V9HW71	ERP29	Endoplasmic reticulum resident protein 29
Q96HE7	ERO1A	ERO1-like protein alpha

**Table 8:** Proteins showing higher abundance in RYR-treated and control cells.

Accession	Gene Names	Protein Names
C9J0K6	SRI	Sorcin
P62854	RPS26	Small ribosomal subunit protein eS26
X1WI28	RPL10	Large ribosomal subunit protein uL16
B7Z992	-	Gelsolin
P02452	COL1A1	Collagen alpha-1(I) chain
A0A384MDU2	COL1A2	Collagen, type I, alpha 2, isoform CRA_c
A0A5F9UP49	SDF4	45 kDa calcium-binding protein

**Table 9:** Proteins showing higher abundance following simvastatin treatment.

Accession	Gene Names	Protein Names
O15031	PLXNB2	Plexin-B2
Q5H9A7	TIMP1	Metalloproteinase inhibitor 1
P10301	RRAS	Ras-related protein R-Ras
P11233	RALA	Ras-related protein Ral-A
A8K401	PHB	Prohibitin
P50570	DNM2	Dynamin-2
A0A087X054	HYOU1	Hypoxia up-regulated protein 1
Q9Y639	NPTN	Neuroplastin

## Data Availability

The original contributions presented in this study are included in the article/Supplementary Material. Further inquiries can be directed to the corresponding author. The mass spectrometry proteomics data have been deposited in the ProteomeXchange Consortium via the PRIDE [20] partner repository with the dataset identifier PXD082271.

## Supplementary Materials

The following supporting information can be downloaded at: https://www.mdpi.com/article/10.3390/foods15162889/s1. See the supplementary material file for untargeted metabolomics analysis, exploratory proteomics analysis, the amount of RYR extract table, and cytotoxicity raw data.
